# Three hydrophobic surface binding a proteins link surface hydrophobicity to sporulation, biofilm formation, and host interaction in *Mucor lusitanicus*

**DOI:** 10.3389/fcimb.2026.1904531

**Published:** 2026-08-20

**Authors:** Anna Molnár, Amanda Grace Vaz, Mónika Homa, Chetna Tyagi, László Bodai, Gábor Nagy, Nóra Zsindely, Dóra Németh, Rita Sinka, Alfonz Kedves, Andrea Rónavári, Zoltán Kónya, László Janovák, Bence Rafael, Botond Ferenc Szegedi, Karina Kiss, Tammam Khaliefeh, Kerstin Voigt, Gábor Nagy, Csilla Szebenyi, Tamás Papp

**Affiliations:** 1 HUN-REN-SZTE Fungal Pathomechanisms Research Group, University of Szeged, Szeged, Hungary; 2 Department of Biotechnology and Microbiology, Faculty of Science and Informatics, University of Szeged, Szeged, Hungary; 3 Department of Biochemistry and Molecular Biology, Faculty of Science and Informatics, University of Szeged, Szeged, Hungary; 4 Department of Genetics, Faculty of Science and Informatics, University of Szeged, Szeged, Hungary; 5 Department of Applied and Environmental Chemistry, Faculty of Science and Informatics, University of Szeged, Szeged, Hungary; 6 Faculty of Agriculture, Institute of Animal Science and Wildlife Management, University of Szeged, Hódmezővásárhely, Hungary; 7 HUN-REN-SZTE Reaction Kinetics and Surface Chemistry Research Group, University of Szeged, Szeged, Hungary; 8 Department of Physical Chemistry and Materials Science, University of Szeged, Szeged, Hungary; 9 Jena Microbial Resource Collection (JMRC), Leibniz Institute for Natural Product Research and Infection Biology – Hans Knoell Institute, Jena, Germany; 10 HUN-REN Biological Research Centre, Institute of Genetics, Szeged, Hungary

**Keywords:** biofilm, HsbA proteins, hydrophobic surface binding, mucormycosis, sporulation, virulence factor

## Abstract

**Introduction:**

*Mucor lusitanicus* is a model organism for studying fungal development and physiology, as well as pathogenicity of Mucorales fungi. Hydrophobic surface-binding proteins (HsbA family) have been described in filamentous fungi as interface-associated factors involved in adhesion, enzymatic recruitment, and surface interactions; however, their functional diversification in Mucorales remains poorly understood.

**Methods:**

Here, we present a comprehensive characterization of three HsbA proteins in *M. lusitanicus*.

**Results:**

All examined HsbA proteins share a conserved α-helical fold with a hydrophobic core and are capable of binding fatty acids, while displaying differential affinity for hydrophobic interfaces.

**Discussion:**

These structural properties translate into distinct surface-associated functions, including modulation of surface hydrophobicity, biofilm formation, and sporangial architecture. Genetic analyses further demonstrate that HsbA proteins play a central role in developmental regulation, affecting spore germination timing, stress responses, sporulation, and spore hydrophobicity. At the host interaction level, HsbA overexpression increases early phagocytic uptake but impairs infection progression, whereas gene disruption enhances virulence in *in vivo* insect models. These findings support a model in which HsbA proteins primarily regulate developmental timing rather than acting as classical virulence determinants. Collectively, our results show that HsbA proteins in *M*. *lusitanicus* function as regulators that couple fungal surface remodeling with developmental transitions. Unlike previously characterized fungal surface systems that mainly mediate adhesion, immune evasion, or enzymatic recruitment, *Mucor* HsbA proteins integrate surface properties with growth timing, thereby coordinating environmental adaptation and host–pathogen interactions.

## Introduction

*Mucor lusitanicus* is a frequently used genetic model organism for studying Mucorales biology, development, and virulence (Lax et al., 2022; Tahiri et al., 2023; Osorio-Concepción et al., 2024; Trieu et al., 2024; Tahiri et al., 2025, 2026). The availability of its complete genome sequence and a broad range of molecular tools (Tahiri et al., 2025), including efficient transformation systems (Gutiérrez et al., 2011), RNA interference-based functional genomics (Cervantes et al., 2013), and CRISPR–Cas9-mediated genome editing (Nagy et al., 2017; Szebenyi et al., 2023), has established *M. lusitanicus* as one of the most genetically tractable representatives of the Mucorales order (Lax et al., 2022). Studies performed in *M. lusitanicus* have provided important insights into fungal dimorphism (Homa et al., 2022), cell wall biogenesis, iron acquisition (Alejandre-Castañeda et al., 2022), stress responses, antifungal resistance, RNA interference mechanisms (Tahiri et al., 2025; Pérez-Arques et al., 2025), and host–pathogen interactions of mucormycosis causing fungi (Gebremariam et al., 2014; Ibrahim and Voelz, 2017). Hydrophobic surface binding protein A (HsbA) represents a conserved family found across diverse fungal taxa, whose evolution is shaped by lineage-specific duplications (notably in Eurotiales and Mucoromycotina), and which function in *Aspergillus* to recruit lytic enzymes to hydrophobic surfaces, suggesting a conserved role in mediating cell wall interactions and enzymatic degradation of extracellular substrates (Muszewska et al., 2017). HsbA protein was first isolated from the culture broth of *Aspergillus oryzae* RIB40 grown in a medium containing polybutylene succinate-co-adipate (PBSA) as a sole carbon source where HsbA was shown to recruit the CutL1 cutinase to polybutylene succinate-co-adipate (PBSA), thereby promoting degradation of this hydrophobic substrate (Ohtaki et al., 2006). Notably, *Aspergillus terreus*, *A*. *flavus*, and *A*. *niger* secrete HsbA proteins that enhance the recruitment of degradative enzymes to hydrophobic substrates such as wheat straw; in *A. niger*, this is accompanied by transcriptional upregulation of hydrophobin genes upon exposure to lignocellulosic material, reflecting a coordinated surface-binding response (Delmas et al., 2012). This recruitment mechanism represents an important feature of HsbA function, contributing not only to interactions with hydrophobic substrates but also to fungal pathogenicity by facilitating host surface colonization and tissue invasion (Aimanianda et al., 2009; Shimizu et al., 2019). Orthologs of the *A. oryzae hsbA* gene have also been identified in the insect pathogenic fungus *Beauveria bassiana*, where HsbA proteins contribute to adhesion to hydrophobic insect cuticles and accumulate during infection, with localization detected at the cuticle penetration site in the infected host *Frankliniella occidentalis* (Zhang et al., 2013). Similarly, in plant-associated fungi, HsbA expression has been associated with host colonization, as demonstrated by the induction of HsbA-encoding genes during early infection stages in *Colletotrichum fructicola* and *Magnaporthe oryzae* (Soanes et al., 2012; Zhang et al., 2018). In *M. oryzae* MoSVP (for *M. oryzae* Secreted Virulence Protein) encodes an HsbA domain-containing protein with a signal peptide at its N-terminus and a sequence with no known similarity to other proteins at its C-terminus (Shimizu et al., 2019). Structural analysis of this protein revealed a similar HsbA domain in the immunogenic Mp1 protein of the opportunistic human pathogen *Talaromyces marneffei* (Shimizu et al., 2019), which suppresses inflammatory responses in the host via its arachidonic acid binding capability by potentially interfering with prostaglandin and leukotriene biosynthesis (Lam et al., 2019).

The aim of this research is to functionally characterize three HsbA proteins and their coding genes for the first time in a fungus belonging to the order Mucorales, and to investigate their role in the physiology and pathogenicity of the fungus.

## Results

### Identification of *hsbA* genes in the *M. lusitanicu*s genome

Eight putative HsbA homologs were identified in the *Mucor lusitanicus* genome, designated as HsbA1a (Protein ID: 1482774), HsbA1b (1482775), HsbA2 (1442756), HsbA3 (1474024), HsbA4 (1432553), HsbA5 (1594666), HsbA6 (1522749) and HsbA7 (1567658). The genomic locations and predicted features of the encoded putative proteins are summarized in **Table 1**. Remarkably, the nucleotide sequences of *hsbA1a* and *hsbA1b* were found to be identical (**Supplementary Figure 1A**), differing only in their promoter and terminator regions (**Supplementary Figure 1B**); moreover, these two genes are located directly adjacent to each other in the genome (**Table 1**). All eight predicted HsbA proteins contain a signal peptide, suggesting their secreted nature (**Table 1**). HsbA domain containing proteins showing >30% sequence identity to *M. lusitanicus* HsbA proteins in other fungi are summarized in **Supplementary Table 2**, showing that HsbA-related proteins are widespread across diverse fungal taxa, including Mucorales, Ascomycota, and Basidiomycota. A sequence similarity-based comparison of proteins showing >30% amino acid sequence identity with *M. lusitanicus* HsbA proteins is shown in **Supplementary Figure 2**. The present study focuses on the characterization of *hsbA1a*, *hsbA1b*, *hsbA2* and *hsbA3* and the encoded proteins of *Mucor*. The HsbA proteins show high similarity to each other, with the highest sequence similarity observed between HsbA1 and HsbA2 (84%), while all pairwise identities exceed 50% (**Table 2**).

**Table 1:** Protein length, molecular weight, genomic location, signal sequence features, and signal sequence cleavage site of HsbA proteins in *Mucor lusitanicus*.

Protein	Length (aa)	Scaffold	Strand	Position	Weight of matured protein (kDa)	Signal sequence location	Signal sequence cleavage site
HsbA1a	185	2	+	4160729-4161414	17.15	between aa 1 to 19	between aa 19 and 20; ANA-AA
HsbA1b	185	2	–	4163635-4164315	17.15	between aa 1 to 19	between aa 19 and 20; ANA-AA
HsbA2	185	1	–	2177228-2177874	17.33	between aa 1 to 19	between aa 19 and 20; AHA-AA
HsbA3	184	2	+	1092205-1092921	16.9	between aa 1 to 18	between aa 18 and 19; VNA-AA
HsbA4	185	2	–	1810997-1811617	17.75	between aa 1 to 18	between aa 18 and 19; VNA-AA
HsbA5	185	2	+	3589528-3590206	17.08	between aa 1 to 19	between aa 19 and 20; ANA-AA
HsbA6	175	5	+	3115601-3116234	18.73	between aa 1 to 19	Between aa 19 and 20; VNA-AA
HsbA7	182	7	–	2829650-2830341	19, 028	between aa 1 to 20	Between aa 20 and 21; VNA-AA

**Table 2:** Pairwise amino acid sequence similarity of HsbA proteins.

Protein	HsbA1a/ HsbA1b	HsbA2	HsbA3	HsbA4	HsbA5	HsbA6	HsbA7
HsbA1a/HsbA1b	–	84%	62%	33%	95%	27%	42%
HsbA2	84%	–	56%	33%	84%	28%	44%
HsbA3	62%	56%	–	45%	61%	29%	37%
HsbA4	33%	33%	45%	–	32%	25%	26%
HsbA5	95%	84%	61%	32%	–	28%	44%
HsbA6	27%	28%	29%	25%	28%	–	22%
HsbA7	42%	44%	37%	26%	44%	22%	–

### *In silico* analysis of structure and hydrophobic surface binding of HsbA1, HsbA2 and HsbA3 proteins

**Figure 1:**
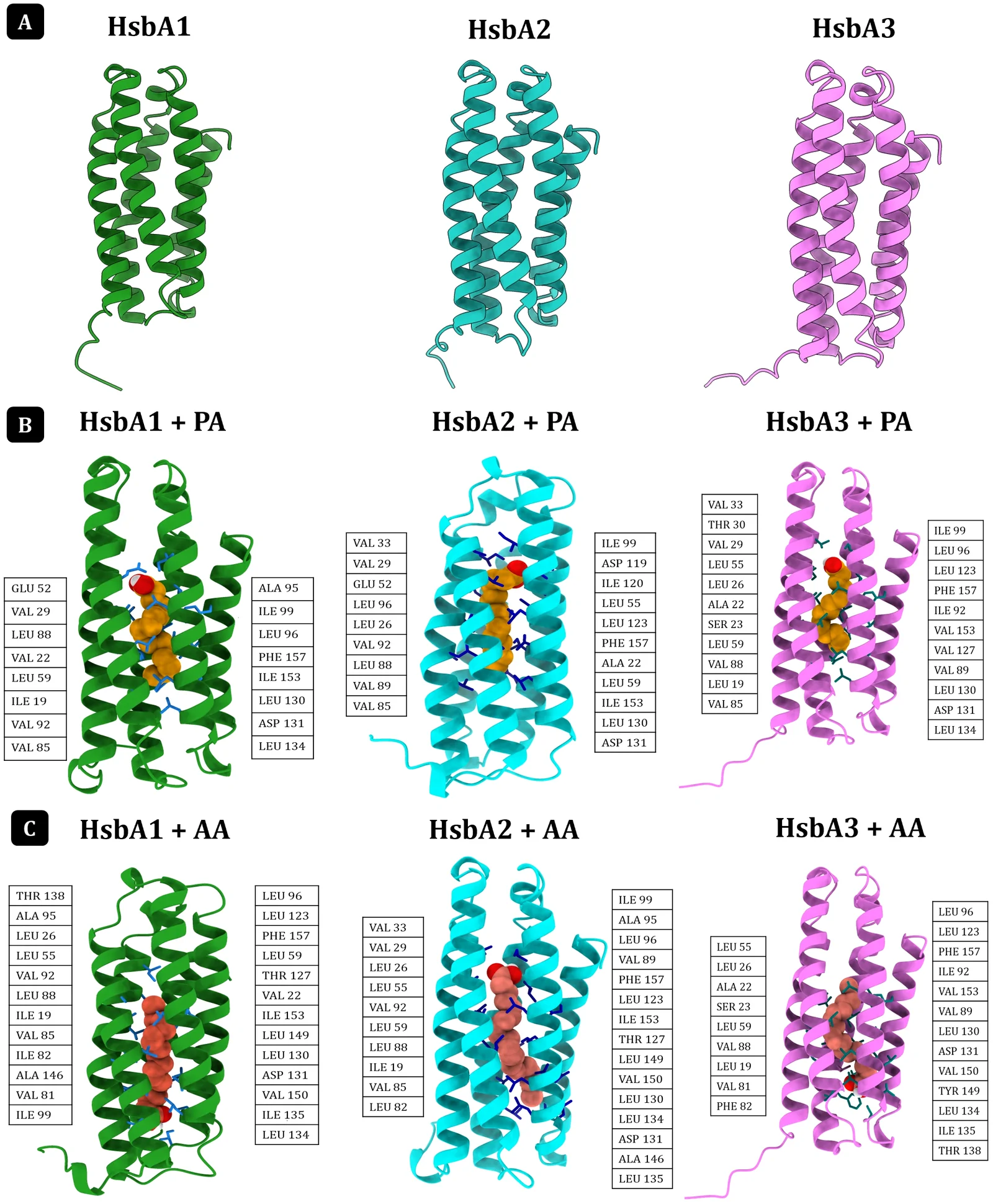
**(A)** Predicted folded structures of HsbA1–3 proteins generated using AlphaFold 3. **(B)** The prediction of the binding of HsbA1–3 proteins to palmitic acid (PA) and arachidonic acid (AA) **(C)** by AutoDock Vina. The tables are listing the amino acids (of which residues are shown on the proteins) and their position that exhibit binding affinity to palmitic acid and arachidonic acid in the context of HsbA1–3 proteins. Green: HsbA1 protein; cyan: HsbA2 protein; magenta: HsbA3 protein; orange: palmitic acid; coral: arachidonic acid; red: oxygen molecule.

**Figure 2:**
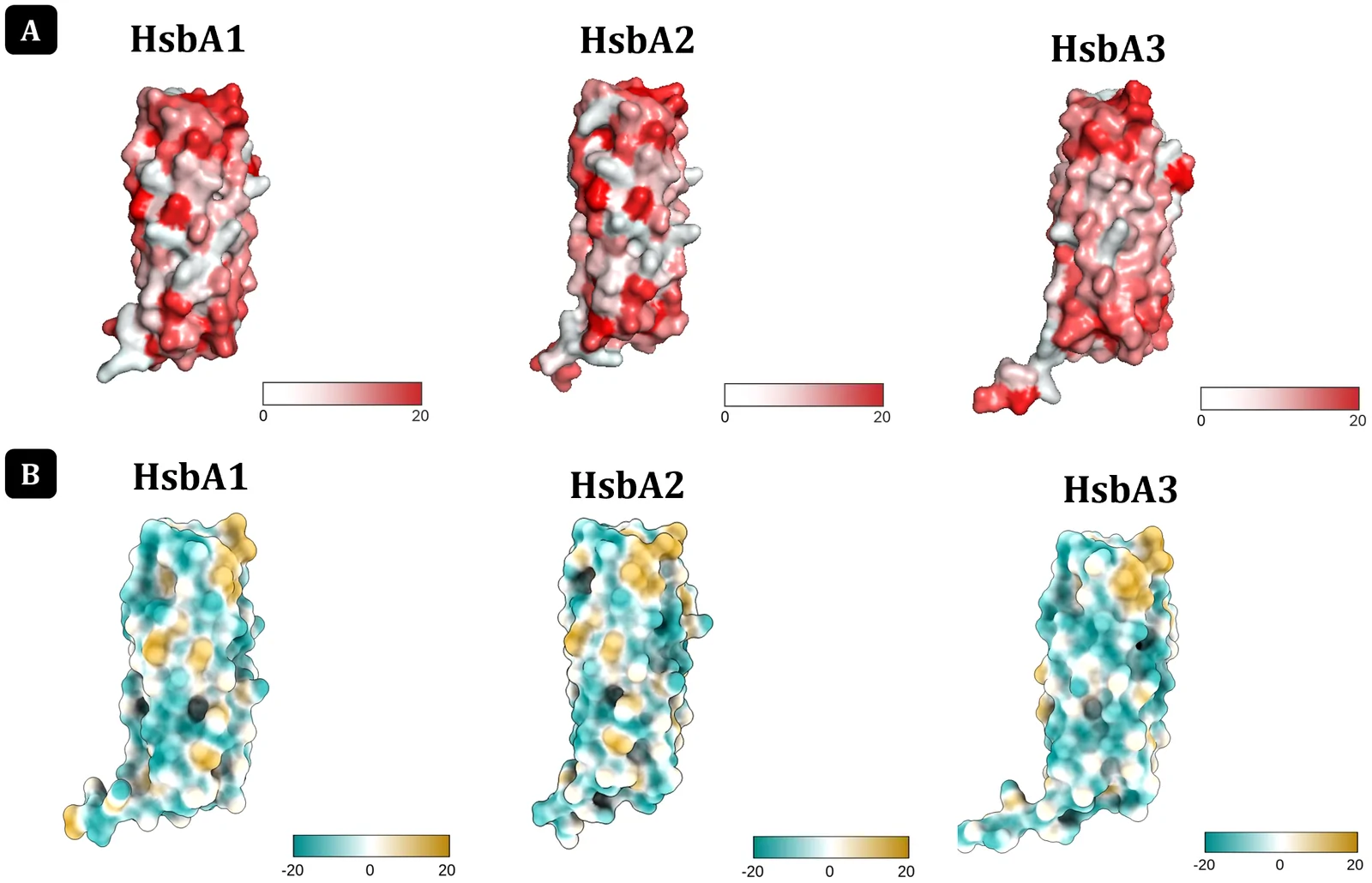
The color scales indicate **(A)** Surface hydrophobicity and **(B)** surface lipophilicity of HsbA1–3. In panel A, white represents the lowest hydrophobicity and red the highest hydrophobicity. In panel **(B)**, blue indicates the lowest lipophilicity, while yellow represents the highest lipophilicity.

**Figure 3:**
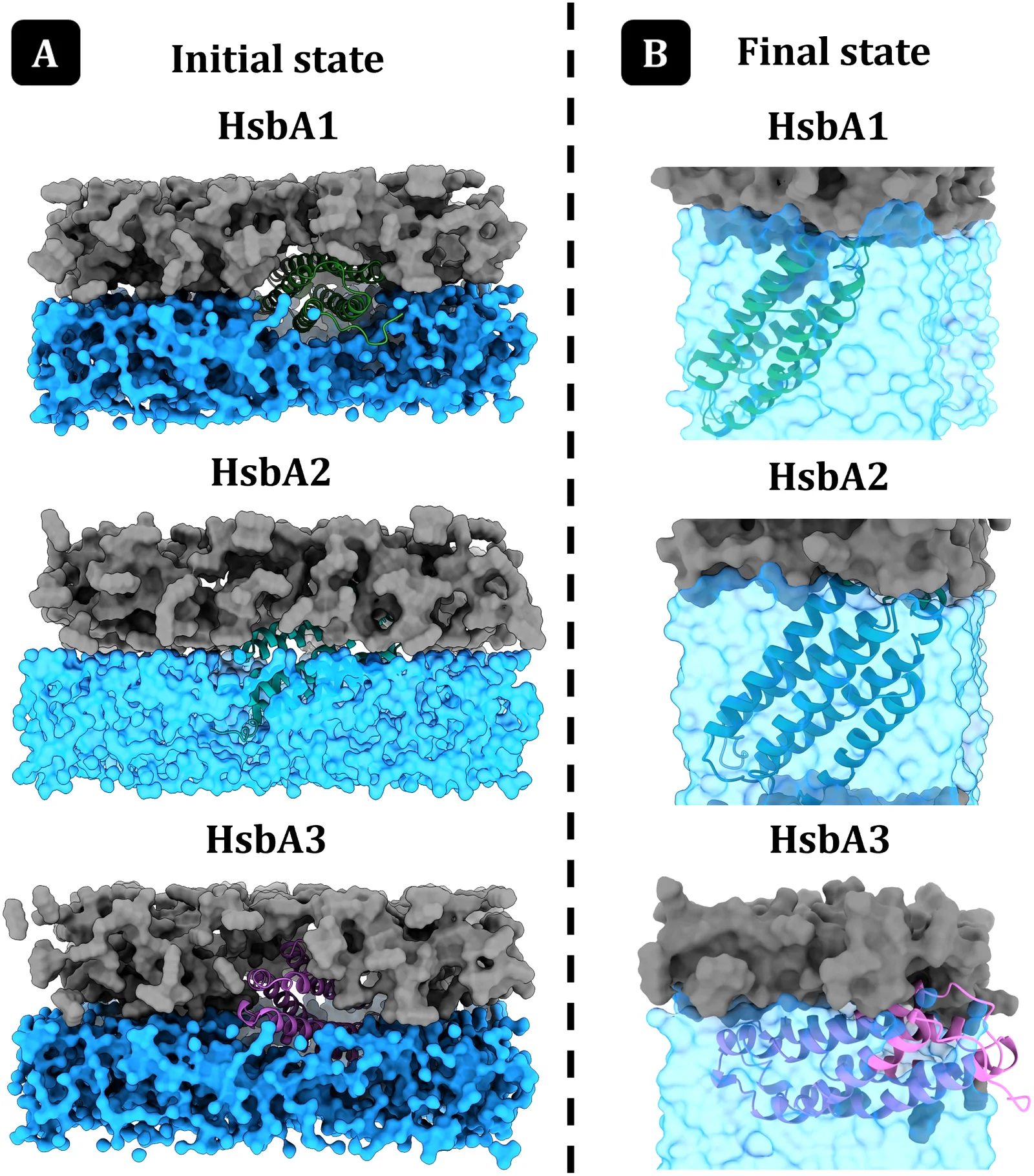
Molecular dynamics simulation of the binding mode of folded proteins, HsbA1–3 proteins, at the interface of water-hexane layers. **(A)** Initial state of HsbA1–3 proteins at water-hexane interface. **(B)** Final state of HsbA1a-3 proteins at water-hexane interface. Gray: hexane layer; Cornflower blue: aqueous layer; Green: HsbA1 protein; Cyan: HsbA2; Magenta: HsbA3 protein.

Using the amino acid sequences of HsbA proteins, we predicted the folded structures using the state-of-art AlphaFold3 where the results revealed quite similar structures for all three proteins with high confidence scores (ipTM) ranging from 0.84 to 0.87, respectively (**Figure 1A**). All of them fold into 5 helices arranged in a way to form a long channel-like cavity. All hydrophobic amino acid residues are faced inwards to the cavity making it a perfect binding site for lipid/fatty acid chains. The closest structural identity of these structures was found with the Mp1 protein (Mp1p) (Lam et al., 2019), a secretory galactomannoprotein antigen of *T. marneffei* with two tandem ligand-binding domains (Mp1p-LBD1 and Mp1p-LBD2) (Pdb ID: 5E7X). The LBD1 domain also folds into 5 helices forming a hydrophobic cavity for binding palmitic acid (PA) and arachidonic acid (AA). Therefore, we carried out docking calculations of PA (**Figure 1B**) and AA (**Figure 1C**) with all HsbAs using AutoDock Vina and also used the latest AI-based co-folding algorithms Boltz-1 and Boltz-2. The results are summarized in **Table 3**. Overall, the results show that both PA and AA could bind to the hydrophobic cavity of all HsbAs with varying degrees of affinity. However, empirical methods of ligand docking like Autodock Vina, scored the binding of AA with HsbA3 as the best amongst all while AI-based co-folding algorithms Boltz-1 and 2 scored PA binding with HsbA1 as the best (**Figures 1B, C**). Moreover, the binding pose of both PA and AA may be in exactly opposite orientation from each other depending on the state of the protein. This observation may be due to how differently the two algorithms approach ligand-binding but there is ample reason to believe that the orientation preference of bound PA and AA is dependent on whether the protein is in open or closed conformation, as also noted by Lam et al. (2019) in case of Mp1p-LBD1 and LBD2 proteins. Furthermore, Ohtaki et al. (2006) explored the characteristics of HsbA produced by *A. oryzae* where it adsorbs onto hydrophobic PBSA surfaces even though the sequence suggests that the outer surface of HsbAs is not highly hydrophobic. It even recruited CutL1 polyesterase and promoted PBSA degradation. Naturally, we were curious whether these HsbAs could show any of such properties. Their surface lipophilicity surfaces are depicted in **Figures 2A, B**. All three HsbAs are mostly hydrophilic (cyan) but all of them have a highly hydrophobic region (red) in form of a loop connecting helices 3 and 4 and containing residues like leucine and isoleucine. This region may be the initial contact point in binding with a hydrophobic surface. To test this hypothesis, we placed fully folded HsbA1, 2 and 3 at the interface of water (cornflower blue) and hexane (gray) molecules and carried out short molecular dynamics (MD) simulations for 50 ns. (**Figure 3**). Quite early in the MD simulation (**Figure 3A**), HsbA1 and 2 re-oriented themselves to completely immerse in the water solvent where the C-terminal hydrophobic patch remains attached to the hydrophobic hexane surface for the entirety of the simulation. Few hexane molecules even entered the hydrophobic cavity of HsbA1 but not in case of HsbA2 as shown in the figure. This behavior gives an insight on how HsbAs may be adsorbed onto a hydrophobic surface via the single patch present at the C-terminal opening. On the other hand, in case of HsbA3, after initial contact with the hexane surface, the whole protein reorients to interact with the hydrophobic phase (**Figure 3B**). Distribution of hydrophilic and hydrophobic surface patches in the final MD states is presented in **Supplementary Figure 3**. The proteins may even be able to recruit fatty acids like PA and AA by binding them and creating an overall hydrophobic exterior.

**Table 3:** Docking calculations of Palmatic Acid (PA) and Arachidonic Acid (AA) with all HsbAs using AutoDock Vina, Boltz-1 and Boltz-2.

Docking calculations	HsbA1	HsbA2	HsbA3
Autodock Vina docking score with PA	-4.872 kcal/mol	- 5.624 kcal/mol	-5.911 kcal/mol
docking score with AA	-5.108 kcal/mol	-5.554 kcal/mol	-7.204 kcal/mol
Boltz-1 Gibbs free binding energy with PA	-7.179 kcal/mol	-7.167 kcal/mol	-5.968 kcal/mol
Boltz-1 with AA	-5.766 kcal/mol	-6.714 kcal/mol	-6.343 kcal/mol
Boltz-2 predicted pIC50 with PA	5.332 kcal/mol	5.134 kcal/mol	4.486 kcal/mol
Boltz-2 predicted pIC50 with AA	4.305 kcal/mol	4.218 kcal/mol	4.624 kcal/mol

### Targeted disruption and overexpression of *hsbA1a*, *hsbA2* and *hsbA3* genes

Single-gene disruptions of *hsbA1a*, *hsbA2*, and *hsbA3* were successfully carried out in *M. lusitanicus* using CRISPR-Cas9-mediated integration of the *pyrG* selection marker gene (Nagy et al., 2017; Song et al., 2019), resulting in the mutant strains MS12-*ΔhsbA1+pyrG*, MS12-*ΔhsbA2+pyrG*, and MS12-*ΔhsbA3+pyrG*, respectively (**Supplementary Figure 2A**). Notably, the *hsbA1* gene exists as two identical coding sequence copies (*hsbA1a* and *hsbA1b*) in the genome, distinguished by variations in their promoter and terminator regions (**Supplementary Figure 1B**). To ensure targeted disruption of *hsbA1a*, the disruption cassette was designed with homologous flanking regions specific to the *hsbA1a* promoter and terminator. Quantitative reverse transcription PCR (qRT-PCR) analysis corroborated the absence of *hsbA1*, *hsbA2*, and *hsbA3* transcripts in the respective mutants, indicating complete gene disruption (**Supplementary Figure 4B**). Notably, qRT-PCR analysis revealed that no *hsbA1* transcript was detectable in the *ΔhsbA1*a mutant. The absence of *hsbA1* transcript was persistent and it did not appear under the tested culture conditions suggesting that the second coding sequence copy, *hsbA1b*, is not transcriptionally active. Whole genome sequencing (WGS) (Smith et al., 2014) of the MS12-*ΔhsbA1a+pyrG* mutant also confirmed that only *hsbA1a* was disrupted, while *hsbA1b* and the adjacent regions remained unaffected. Our results indicate that *hsbA1b* is transcriptionally silent, at least under the tested conditions. WGS analysis of the MS12-*ΔhsbA2+pyrG* mutant similarly confirmed the specific disruption of *hsbA2*. The sequencing data is available in the NCBI SRA database (NCBI SRA database ID: PRJNA1463750). Overexpression of *hsbA1a*, *hsbA2*, and *hsbA3* in *M. lusitanicus* was achieved via transformation with circular plasmids pAV1, pAV2, and pAV3, yielding the strains MS12+pAV1, MS12+pAV2, and MS12+pAV3, respectively (**Supplementary Figure 4C**). qRT-PCR analysis demonstrated significantly elevated transcript levels of *hsbA1a*, *hsbA2*, and *hsbA3* in their respective overexpression mutants compared to the parental MS12 strain, with fold changes reaching statistical significance (*p ≤ 0.05–0.01; **p ≤ 0.001–0.0001) (**Supplementary Figure 4D**). By employing *hsbA1*-specific primers capable of differentiating the two *hsbA1* gene copies based on sequence divergence in the region upstream of the start codon, we confirmed the successful disruption of the targeted *hsbA1a* gene and the intact state of *hsbA1b* (**Supplementary Figure 4E**; **Supplementary Table 1**) in all mutants.

### Transcription patterns of *hsbA1a*, *hsbA2* and *hsbA3* genes

**Figure 4:**
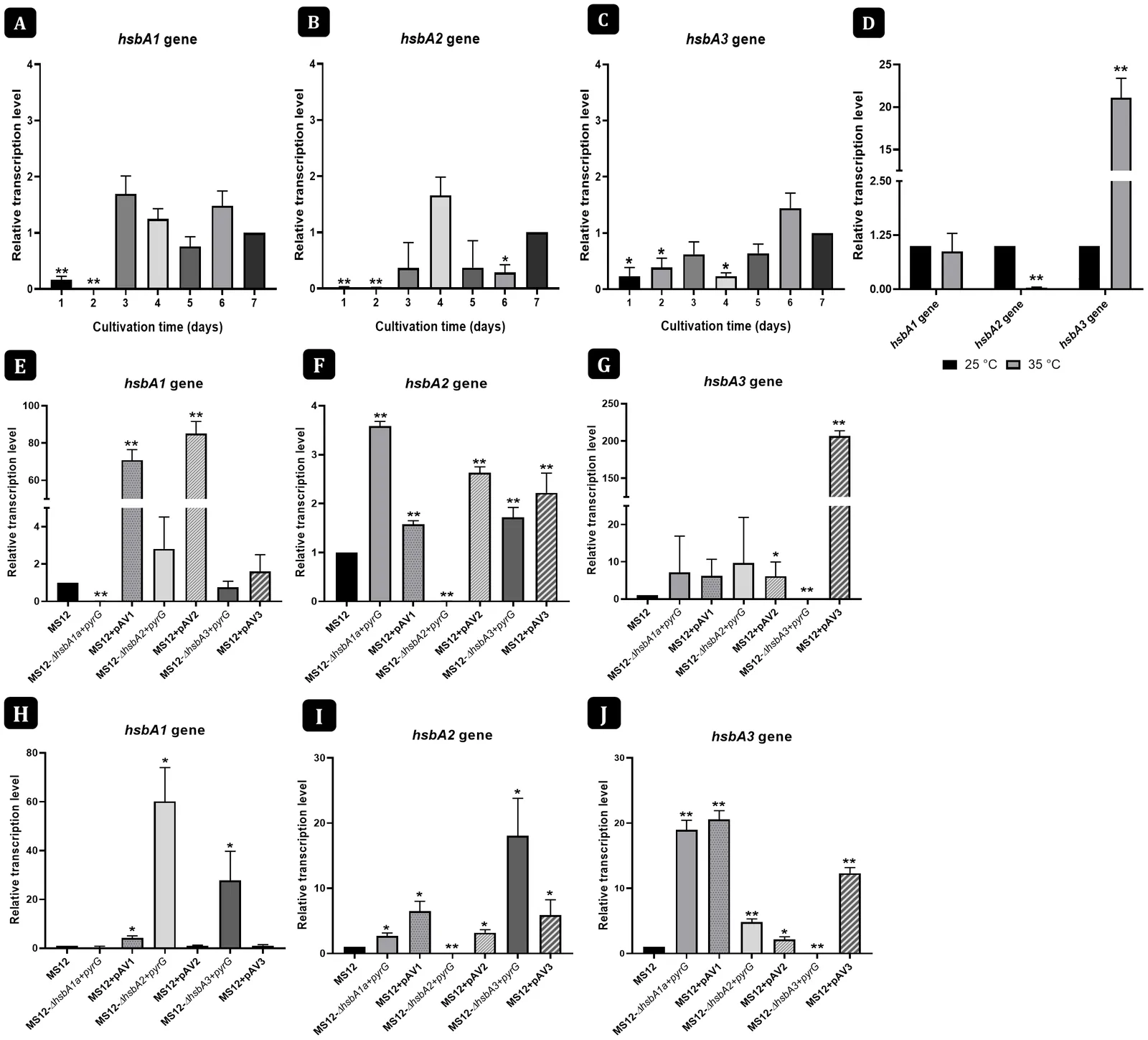
Gene expression levels of the *hsbA1–3* genes **(A–C) i**n the parental MS12 strain cultivation on YNB+L medium at 25 °C for 1–7 days. Significance was calculated using an unpaired two-sample t-test relative to the relative transcription level measured on day 7, where *p ≤ 0.05-0.01 and **p≤ 0.001-0.0001. **(D)** Relative transcript levels of *hsbA1–3* genes in parental MS12 strain at 4th day of cultivation on YNB minimal medium at 25 °C and 35 °C. **(E–G)** Relative transcription levels of the *hsbA1–3* genes in *hsbA* disruption and overexpression mutant strains at 4th day of cultivation on YNB minimal medium at 25 °C. **(H–J)** Relative transcript levels of *hsbA1–3* genes in *hsbA* disruption and overexpression mutant strains at 4th day of cultivation on YNB minimal medium at 35 °C. Significance was determined using a two-sample unpaired t test relative to the parental MS12 strain, where *p ≤ 0.05-0.01; **p ≤ 0.001-0.0001. Error bars on the graph indicate the standard deviation (SD). Error bars on the graph indicate the standard deviation (SD).

The expression patterns of the genes in the parental strain MS12 were analysed over a 7-day cultivation period on minimal medium at 25 °C, with gene expression levels normalized to day 7 (**Figure 4**). All tested *hsbA* genes exhibited low expression levels on the first two days of cultivation (**Figures 4A–C**) at 25 °C (RT). The temporal expression profiles revealed gene-specific patterns. *HsbA1a* displayed increased expression beginning on day 3 of cultivation (**Figure 4A**); *hsbA2* exhibited a marked peak in transcript abundance on day 4 (**Figure 4B**). In contrast, *hsbA3* reached its maximal expression level on day 6 (**Figure 4C**). Growth of the fungus at 35 °C (**Figures 4H–J**), as compared to RT, resulted in differential expression of *hsbA* genes: *hsbA2* was significantly downregulated, whereas *hsbA3* was upregulated (**Figure 3D**). Relative transcript levels of *hsbA* genes in *hsbA* disruption and overexpression strains were analysed by qPCR, where the *hsbA1a* and *hsbA3* genes exhibited a significant upregulation upon overexpression of the *hsbA2* (**Figures 4E, G**). Disruption or overexpression of the two other *hsbA* genes consistently led to overexpression of the *hsbA2* gene (**Figure 4F**). The function of *hsbA3* appeared to be the most independent from that of the others, as its expression elevated only in response to the overexpression of *hsbA2* (**Figure 4G**). Elevated temperature had no effect on *hsbA1a* gene expression in the original MS12 strain (**Figure 4D**). However, in the mutants, a clear temperature-dependent effect can be observed. The *hsbA1a* gene is upregulated exclusively at elevated temperature and only in the absence of *hsbA2* and *hsbA3* (**Figure 4H**). Under elevated temperature conditions, *hsbA2* remained upregulated in response to altered expression levels of the other *hsbA* genes, consistent with the transcription pattern observed at standard temperature (**Figure 4I**). In contrast, *hsbA3*, which displayed only modest upregulation under normal conditions in strains lacking or overexpressing other *hsbA* genes, was markedly induced upon temperature increase (**Figure 4J**).

### Effect of disruption and overexpression of *hsbA1a*, *hsbA2* and *hsbA3* on sporulation, spore germination, spore size distribution

**Figure 5:**
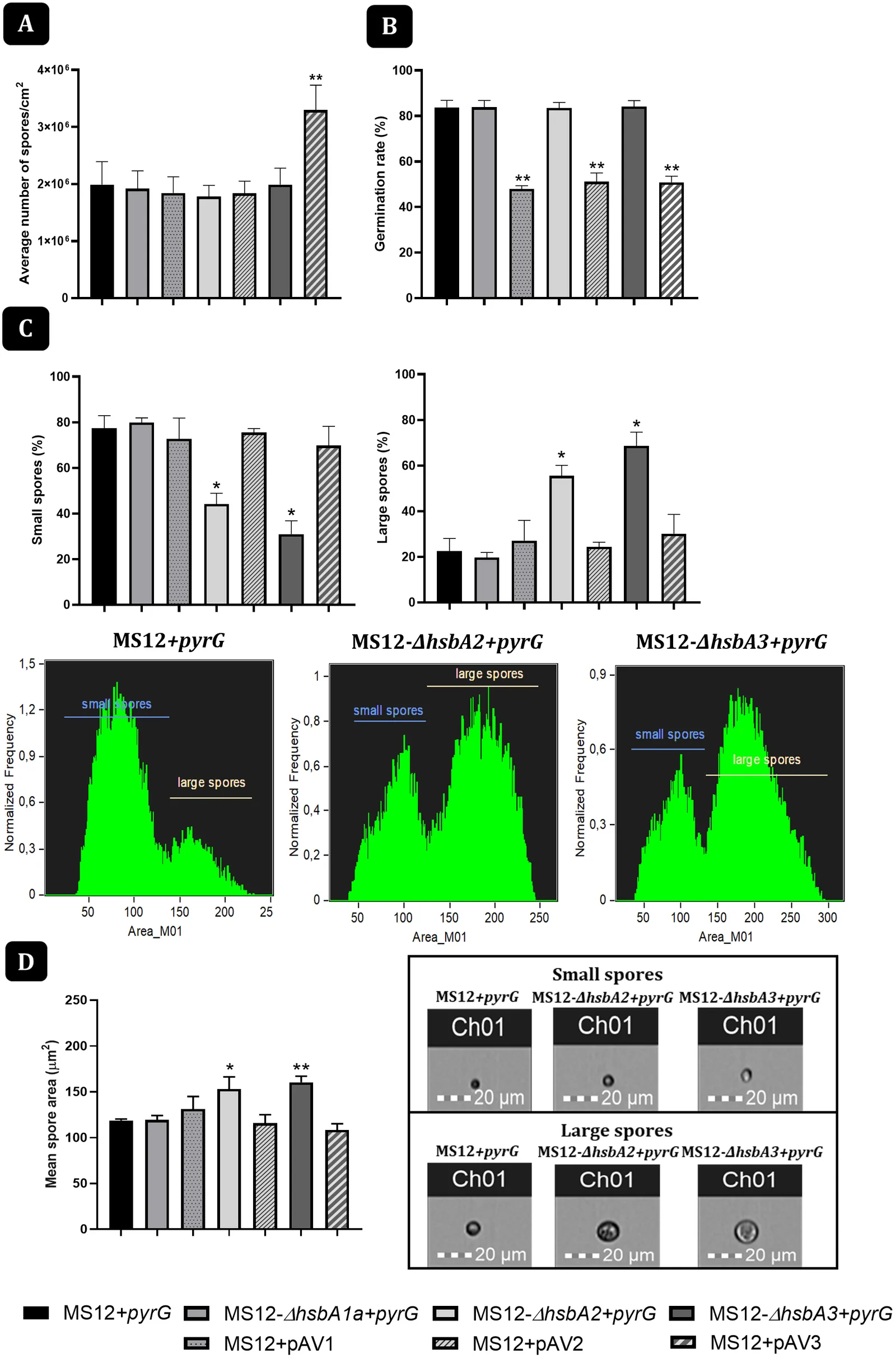
Parameters of spores from *hsbA* mutant strains. **(A)** Quantitative assessment of the sporulation efficiency in *hsbA* mutant strains. **(B)** Germination potential of spores from *hsbA* mutant strains after 8 hours of incubation in YNB+L liquid media. **(C)** The ratio of small and large spores of *hsbA* mutants. Significantly different mutant strains’ (MS12-*ΔhsbA2*+*pyrG*, MS12-*ΔhsbA3*+*pyrG*) and the control strain’s normalized spore size distribution are shown. **(D)** Distribution of the total spore population by mean spore area. The small and large spores of the control and significantly different mutant strains (MS12-*ΔhsbA2*+*pyrG*, MS12-*ΔhsbA3*+*pyrG*) are shown. Spore size was analysed after staining with 5 mM Calcofluor White (CFW) using a FlowSight imaging flow cytometer (Amnis ImageStream X Mk II), and analyzed in IDEAS 6.2. Error bars on the graph indicate the standard deviation (SD). Values indicated by asterisks are significantly different from the value of the MS12+*pyrG* strain according to two-sample unpaired t test: *p ≤ 0.05-0.01; **p ≤ 0.001-0.0001.

Sporulation capacity of *hsbA1a* and *hsbA2* disruption mutant strains was found to be similar to the control strain (MS12+*pyrG*) (**Figure 5A**). However, the sporulation capacity of the *hsbA3* overexpression mutant increased (**Figure 5A**). Moreover, reduced germination capacity was observed in all overexpression strains (*i.e.*, MS12+pAV1, MS12+pAV2 and MS12+pAV3, respectively) (**Figure 5B**). Flow cytometric analysis also revealed that the absence of HsbA2 and HsbA3 proteins resulted in a significant increase in overall spore size. This effect was due to a shift in the size distribution, with fewer small spores and a higher proportion of large spores (**Figure 5C**), leading to an apparent increase in average spore size (**Figure 5D**).

### Effect of disruption and overexpression of *hsbA1a*, *hsbA2* and *hsbA3* on growth, hyphal morphology, and temperature sensitivity

**Figure 6:**
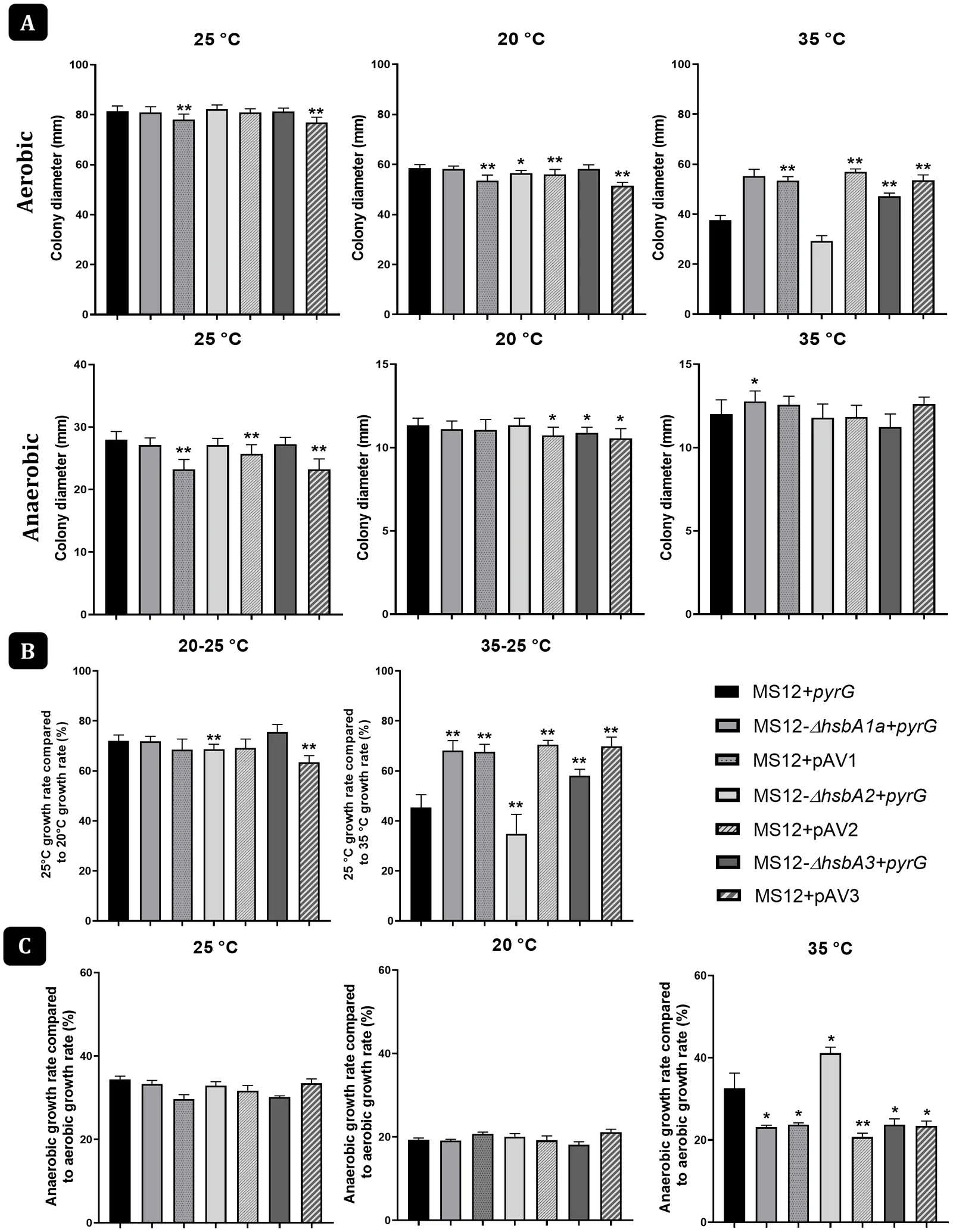
Growth behavior of *hsbA* mutants after 4 days of cultivation in different environmental settings. **(A)** Colony diameters of *hsbA* mutants on minimal medium under aerobic and anaerobic conditions at different temperatures (25 °C; 20 °C; 35 °C) on the 4th day of cultivation. **(B)** Growth rate in percentages (%) cultivated at different temperatures (25 °C; 20 °C; 35 °C) on the 4th day of cultivation compared to growth rate at 25 °C. **(C)** Growth rate in percentages (%) in hypoxia compared to aerobic conditions at different temperatures (25 °C; 20 °C; 35 °C) on the 4th day of cultivation. Values indicated with asterisks are significantly different from the corresponding value of the MS12+*pyrG* strain according to two-way ANOVA per days: *p ≤ 0.05-0.01; **p ≤ 0.001-0.0001. Error bars indicate standard deviation (SD).

The mutants exhibited a consistent growth trajectory from the onset of measurement (**Supplementary Figures 5A, B**); however, herein we focus on the data obtained at day 4. The absence of HsbA proteins did not affect the growth rate or the colony morphology at room temperature (25 °C) compared to the control strain (MS12*+pyrG*) under aerobic conditions (**Supplementary Figure 6**). Overexpression of *hsbA1a* and *hsbA3* resulted in smaller colony sizes under aerobic conditions, and this pattern was not significantly altered under anaerobic conditions (**Figure 6A**). All overexpression mutants displayed altered colony morphology at 25 °C in hypoxia, characterized by more compact growth (**Supplementary Figure 7**). However, under aerobic conditions, the growth of the *hsbA2* and *hsbA3* disruption mutants exhibited a slight decline at 20 °C, with a statistically significant growth reduction observed on day 4 of the measurement (**Figures 6B, C**). In contrast, with the exception of the *hsbA2* disruption mutant strain, mutations in any of the *hsbA* genes led to enhanced growth at 35 °C (**Figure 6B**; **Supplementary Figure 6**). In the case of MS12-*ΔhsbA2+pyrG*, an increase in temperature resulted in a pronounced growth defect under aerobic conditions, whereas under anaerobic conditions at the same temperature, it induced a markedly enhanced growth (at 35 °C) (**Figure 6C**).

### Effect of different stressors on *hsbA1a*, *hsbA2* and *hsbA3* disruption and overexpression mutants

To explore the role of the examined *hsbA* genes in stress responses, we assessed the growth of *M. lusitanicus* control strain (MS12*+pyrG*), three *hsbA* disruption mutants (MS12-*ΔhsbA1a+pyrG*, MS12-*ΔhsbA2+pyrG*, MS12-*ΔhsbA3+pyrG*), and their corresponding overexpressed strains (MS12+pAV1, MS12+pAV2, MS12+pAV3) under various stress conditions. Colony diameters were measured daily over four days at 25 °C in the dark, using Congo Red (CR), Calcofluor White (CFW), SDS, and NaCl as stress agents. We present the measurements from day 4, which reflect the overall growth trend of the mutants. Under CR and CWF-induced stress (**Supplementary Figures 8**, **9**), manipulation of *hsbA* gene expression, either through disruption or overexpression, generally led to increased resistance, with the sole exception of MS12+pAV1 and MS12-*ΔhsbA2+pyrG*, which failed to elicit such an effect. Under SDS-induced membrane stress (**Supplementary Figures 8**, **9**), MS12-*ΔhsbA2+pyrG* and MS12+pAV3 mutants exhibited statistically significant growth differences compared to the control, where disruption of *hsbA2* and overexpression of *hsbA3* reduced colony diameters. Exposure to NaCl stress resulted in significantly impaired growth in MS12+pAV2 and MS12+pAV3 overexpression strains; a similar trend was observed in the *hsbA1* overexpression strain, though the reduction did not reach statistical significance (**Supplementary Figure 9**).

### Role of HsbA1, HsbA2 and HsbA3 proteins in biofilm formation and surface hydrophobicity

**Figure 7:**
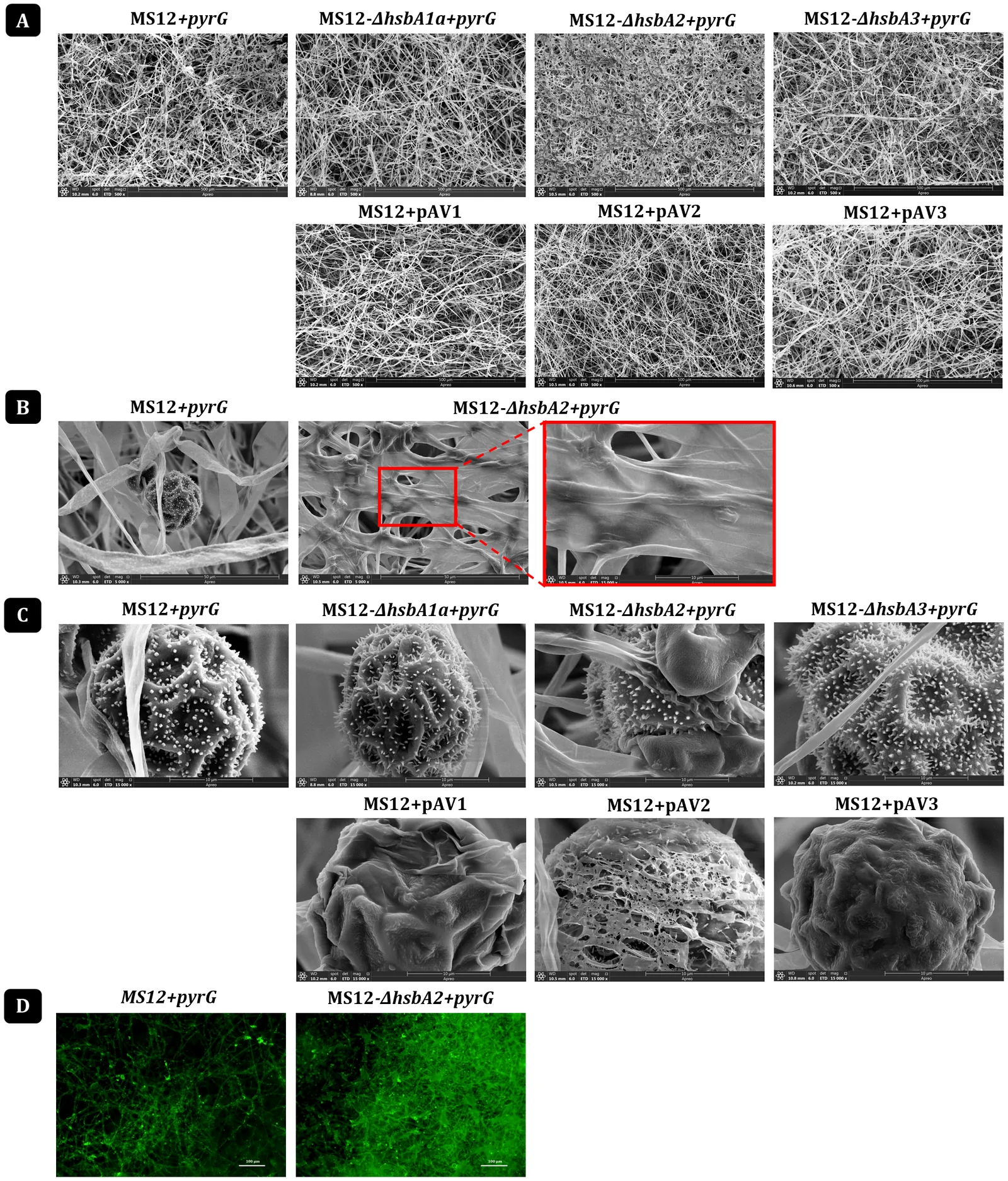
Morphological and structural characterization of *hsbA* mutant mycelia and sporangia by scanning electron (SEM) and fluorescence microscopy. **(A)** Mycelial structure of *hsbA* mutant strains examined by SEM. Images of the MS12+*pyrG* control and *hsbA* mutant strains were taken after 7 days of cultivation at 500× magnification. **(B)** Mycelial structure of the MS12+*pyrG* control strain and the MS12-*ΔhsbA2*+*pyrG* mutant. The red box indicates an extracellular matrix–like structures (ECM) observed in the MS12-*ΔhsbA2*+*pyrG* mutant. Images were acquired at 5000× and 15000× magnifications. **(C)** Surface structure of sporangia from the MS12+*pyrG* control and *hsbA* mutant strains after 7 days of cultivation, imaged at 15000× magnification. **(D)** Mycelial structure of the MS12-*ΔhsbA2*+*pyrG* mutant and MS12+*pyrG* control strain visualized by fluorescence microscopy. Samples were stained with Alexa FluorTM 488 conjugated concanavalin A (ConA-FITC) after 7 days of cultivation. Images were acquired at 10× magnification; scale bars represent 100 µm. Microscopic analyses were carried out with three biological replicates, each with two technical replicates.

**Figure 8:**
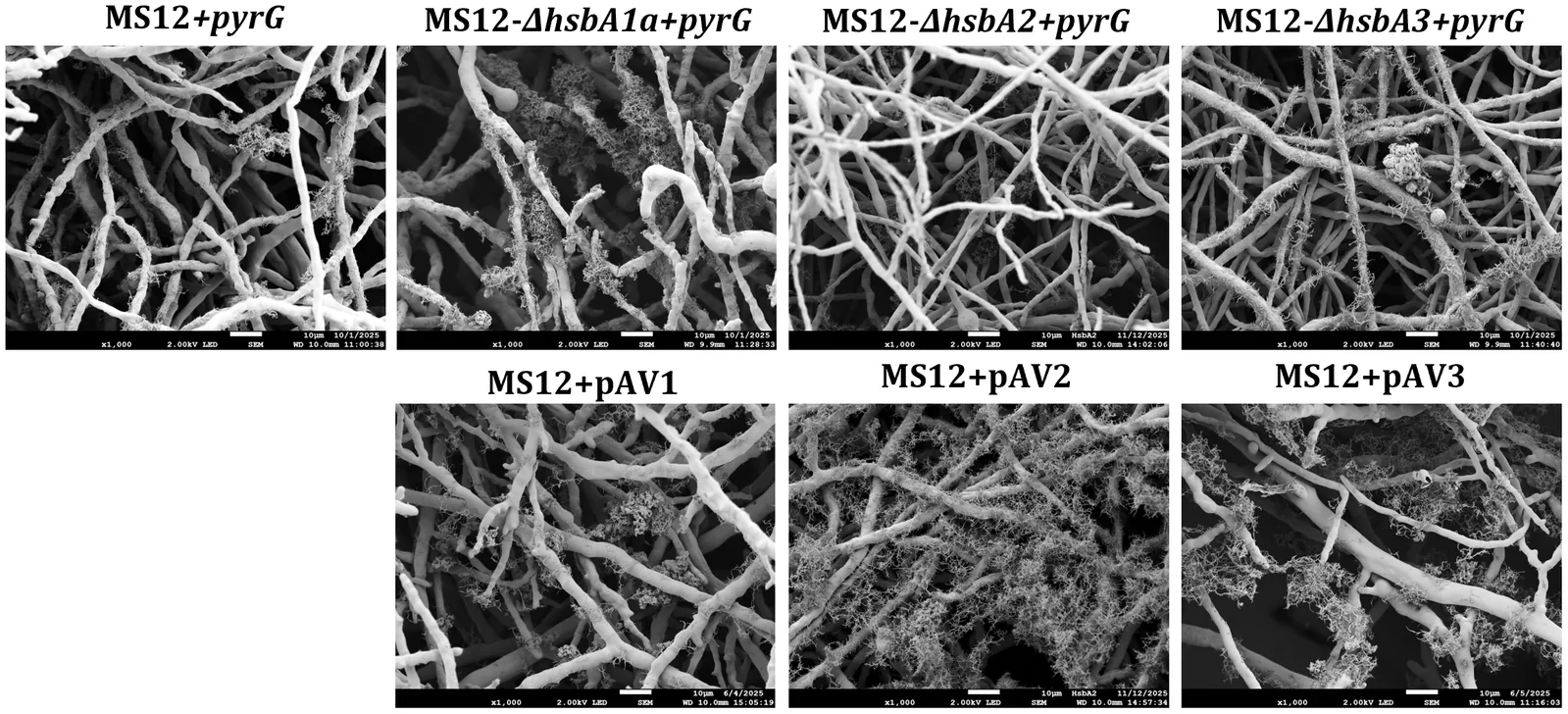
Analysis of biofilm formation in *hsbA* mutant strains utilizing scanning electron microscopy. Structure of the extracellular matrix (ECM) formed by MS12+*pyrG* control and *hsbA* mutant strains after 48 h of incubation. Microscopic images were taken at 2000x magnification.

**Figure 9:**
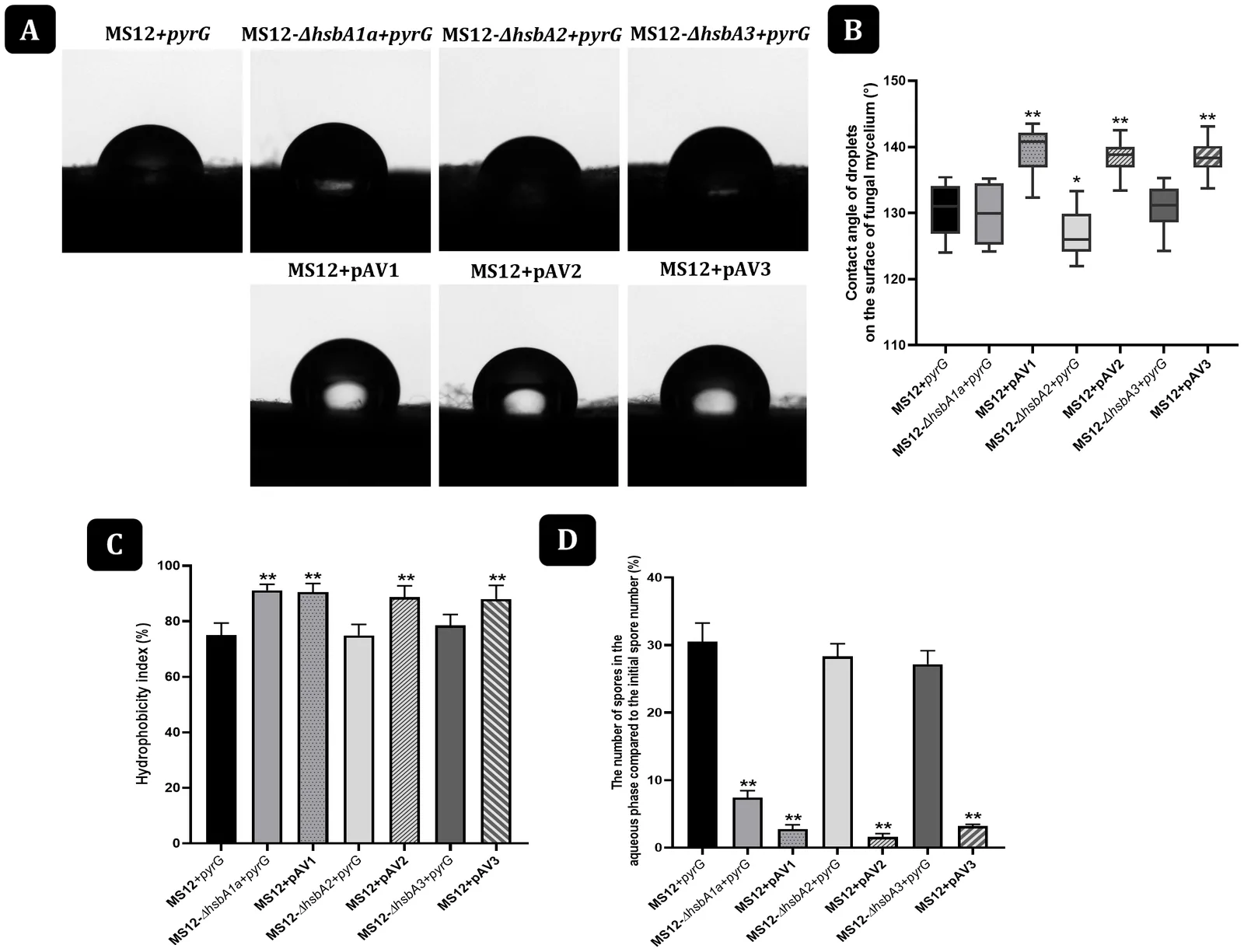
Investigation of cell surface hydrophobicity of *hsbA* mutant strains using contact angle measurement and MATH assay. **(A)** A drop of sterile distilled water on the surface of the mycelium of *hsbA* mutant strains. **(B)** Sessile drop images of water droplets on the surface of fungal mycelium of *hsbA* mutant strains (°). **(C)** Hydrophobicity index (%) of *hsbA* mutant spores form the MATH assay. **(D)** The number of spores in the aqueous phase compared to the initial spore number (%). Values indicated with asterisks are significantly different from the corresponding value of the MS12+*pyrG* strain according to two-sample unpaired t test: **p ≤ 0.001-0.0001. Error bars indicate standard deviation (SD).

Scanning electron microscopy (SEM) of the MS12-*ΔhsbA2+pyrG* revealed altered mycelial surface morphology (**Figure 7A**), with an extracellular matrix (ECM)-like network forming a dense layer covering the hyphal surface (**Figure 7B**). SEM analysis also revealed altered sporangial morphology in the *hsbA* mutants (**Figure 7C**). At day 7, the sporangia of the overexpression strains displayed altered morphology compared to the control, notably lacking the characteristic external spiny layer normally observed at this developmental stage, suggesting a change in sporangial maturation. In MS12-*ΔhsbA2+pyrG*, the sporangia were covered by an above-mentioned extracellular matrix-like material (**Figure 7B**), yet they exhibited maturation and morphology comparable to the control. Concanavalin A-Fluorescein isothiocyanate (ConA-FITC) staining of the sporangiophore-bearing mycelia revealed increased fluorescence intensity in the MS12-*ΔhsbA2+pyrG* mutant strain, suggesting alterations in the composition and/or accessibility of mannose- and glucose-containing cell wall components (**Figure 7D**) (Beauvais et al., 2007). To further investigate this phenotype, we assessed the biofilm-forming capacity of the strains and employed fluorescent staining methods to characterize the nature of the observed extracellular structures. In the overexpression strains, extensive and dense biofilm formation was observed between the hyphae, whereas in MS12*-ΔhsbA2+pyrG*, the biofilm was sparse and loosely organized (**Figure 8**). As these observations did not explain the phenotypic differences in hyphal morphology, we considered that HsbA proteins might alter surface hydrophobicity and therefore assessed the hydrophobicity/wettability of the mycelia using contact angle measurements (**Figures 9A, B**). Loss of HsbA1a or HsbA3 had no significant effect on mycelial hydrophobicity. In contrast, overexpression of any of the three HsbA proteins increased surface hydrophobicity, whereas disruption of *hsbA2* caused a dramatic reduction (**Figure 9B**) (Reza et al., 2016). We further observed that mycelial surfaces are hydrophilic prior to sporulation, and the increase in hydrophobicity occurs only after spore formation, indicating that the presence of spores is a major determinant of mycelial surface hydrophobicity (**Figures 9C, D**).

### Host–pathogen interactions and virulence-associated effects of *hsbA* mutants

#### Interaction of hsbA1a, hsbA2 and hsbA3 disruption and overexpression mutants with macrophage and keratinocyte cells

**Figure 10:**
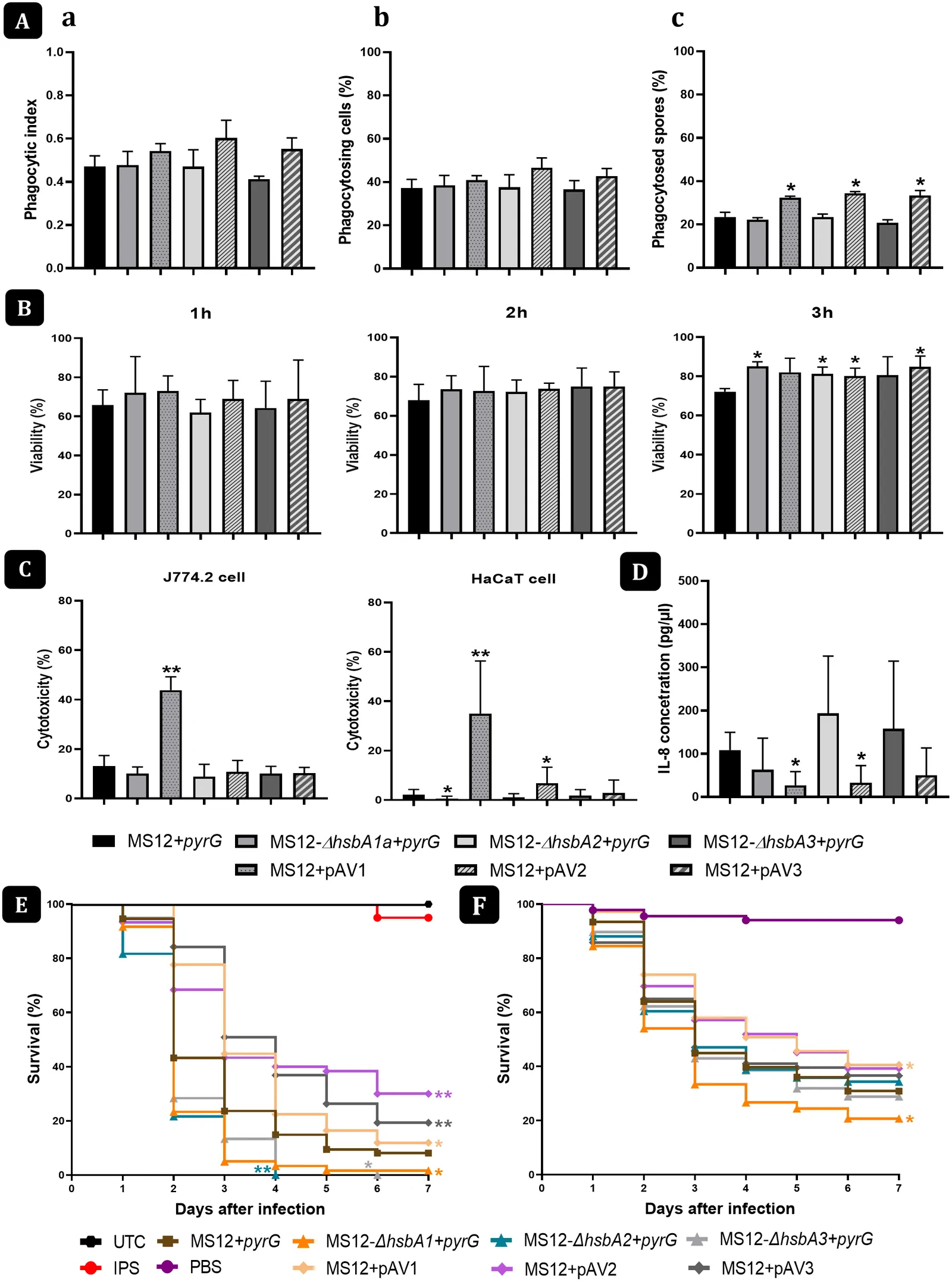
*In vitro* interactions and *in vivo* virulence studies of *hsbA* mutants. **(A)** a, Phagocytic index values; b, percentage of J774.2 cells phagocytosing *hsbA* mutant spores; **c,** ratio of all internalized spores by J774.2 cells. **(B)** XTT metabolic activity assay measured at different time points (1, 2, and 3 h) of infection of J774.2 macrophages with YNB+L pre-incubated spores for 3 h. Absorbance of formazan was measured at 475 nm with background subtraction at 650 nm. **(C)** Cytotoxicity analysis of J774.2 macrophages and HaCaT keratinocytes co-incubated with *Mucor* spores. J774.2 cells (10^5^/mL) were co-incubated with spores (5 × 10^5^/100 µL) for 16 h, whereas HaCaT cells (10^5^ cells/mL) were co-incubated for 30 h. Cytotoxicity was assessed using an LDH assay. Absorbance was measured at 490 nm with background subtraction at 680 nm. **(D)** IL-8 production by HaCaT cells (pg/mL) after 30 h of interaction with *hsbA* mutant spores. HaCaT cells were incubated with spores at a 1:5 cell-to-spore ratio. As a control, HaCaT cells were incubated in the absence of fungi. IL-8 concentrations in culture supernatants were measured using DuoSet ELISA kits (R&D Systems). Cytokine concentrations were calculated based on standard curves generated using a four-parameter logistic curve-fitting model. **(E)** Survival of *Galleria mellonella* (n = 20) infected with *hsbA* mutants and the control *M. lusitanicus* MS12+*pyrG* strain. **(F)** Survival of *Drosophila melanogaster* (n = 60) infected with *hsbA* mutants and the control *M. lusitanicus* MS12+*pyrG* strain. Results represent three independent experiments and are presented as mean ± standard deviation (SD). Statistical significance compared to the MS12+*pyrG* control was determined using a two-sample unpaired t test (*p ≤ 0.05–0.01; **p ≤ 0.001–0.0001) for **(A–D)**, and the Mantel–Cox log-rank test for survival analyses (*p ≤ 0.05–0.01; **p ≤ 0.001–0.0001) for **(E, F)**.

Phagocytosis assays carried out by J774.2 murine macrophages revealed no significant differences in the phagocytic index among the strains (**Figures 10Aa, b**); however, a higher number of spores were internalized in case of the overexpression strains (**Figures 10A, C**). The phagocytosis experiments were complemented with an XTT assay (**Figure 10B**). Spores were incubated for 3 hours (non-germinated), and their metabolic activity was monitored over time using the XTT assay (**Figure 10 B**). During the first 2 hours of interaction, no differences in spore survival were observed among the tested strains. At 3 hours post-phagocytosis, a slight increase in survival was detected in the MS12-*ΔhsbA1*+*pyrG*, MS12-*ΔhsbA2*+*pyrG*, MS12+pAV2, and MS12+pAV3 strains (**Figure 10B**). Based on the LDH cytotoxicity assay, a cytotoxic effect was observed in case of the MS12+pAV1 overexpression strain on macrophages (J774.2) after 16 h of interaction (**Figure 10C**). Considering that macrophage-based assays typically capture early host-pathogen interactions (Wellington et al., 2014), we further evaluated cytotoxicity in a keratinocyte cell line (HaCaT) that tolerates longer incubation periods. After 30 h of incubation, overexpression of HsbA1 resulted in a significant increase in cytotoxicity toward keratinocytes (**Figure 10C**). Besides the MS12+pAV1 overexpression strain, the MS12+pAV2 strain also exhibited significantly increased cytotoxicity, whereas the *hsbA1a* disruption strain showed reduced cytotoxic effects compared to the control (**Figure 10C**). ELISA revealed a marked decrease in Interleukin 8 (IL-8) production by HaCaT keratinocytes upon exposure to the pAV1 and pAV2 mutant strains (**Figure 10D**).

#### Virulence of hsbA1a, hsbA2 and hsbA3 disruption and overexpression mutants in non-vertebrate animal models

In *Galleria mellonella* (**Figure 10E**), strains overexpressing the tested *hsbA* genes exhibited significantly reduced virulence compared to the controls, whereas disruption of *hsbA1a*, *hsbA2*, or *hsbA3* resulted in increased virulence. In the *Drosophila* model (**Figure 10F**), disruption of *hsbA1a* markedly elevated virulence, while overexpression of the same gene led to a substantial reduction in virulence (**Figures 10E, F**).

## Discussion

Hydrophobic surface-binding proteins such as HsbA are widespread in filamentous fungi, including *Aspergillus* and *Cladosporium* species, where they mediate adhesion to hydrophobic substrates and recruit extracellular enzymes to insoluble polymers (Ohtaki et al., 2006; Garrido et al., 2012; Zhang et al., 2018). HsbA-like proteins are also upregulated during early infection in several plant pathogens and are associated with host interaction and surface colonization (Soanes et al., 2012; Zhang et al., 2018). Structural studies show that HsbA-related proteins bind hydrophobic ligands such as fatty acids and mediate interactions at hydrophobic interfaces (Lam et al., 2019). However, their evolutionary diversification and roles in early-diverging fungi such as Mucorales, which include important opportunistic pathogens causing mucormycosis, remain unclear. Here, we provide the first functional characterization of three HsbA proteins in *Mucor lusitanicus* and show that this protein family is expanded and functionally diverse.

We identified eight HsbA-domain proteins in *M. lusitanicus*, all encoding predicted secreted proteins, including a nearly identical duplicate pair (*hsbA1a* and *hsbA1b*) with identical coding sequences but distinct regulatory regions. The presence of these proteins in *M. lusitanicus* and homologous proteins across Mucorales, Ascomycota, and Basidiomycota indicate that HsbA represents a conserved fungal protein family. The high similarity among *M. lusitanicus* HsbA paralogues and their phylogenetic clustering suggest lineage-specific expansion through gene duplications. Despite sequence divergence, HsbA1, HsbA2, and HsbA3 share a conserved five-helix architecture with a hydrophobic cavity, similar to the lipid-binding HsbA-like protein Mp1p from *Talaromyces marneffei* (Lam et al., 2019), supporting the conservation of their potential role in hydrophobic surface and lipid-associated interactions. Under the tested conditions, only *hsbA1a* was expressed, suggesting a regulatory divergence after gene duplication. Asymmetric expression among closely related or identical paralogs is common in fungal gene families and is typically linked to regulatory divergence leading to functional specialization rather than redundancy (*e.g.*, hydrophobins and cutinase gene families) (Karlsson and Stenlid, 2008; Mgbeahuruike et al., 2013).

Structurally, all three HsbA proteins share a conserved α-helical fold forming a channel-like hydrophobic cavity, similar to fungal lipid-binding proteins such as Mp1p from *Talaromyces marneffei* (Cao et al., 1998; Lam et al., 2019). Since Mp1p-mediated lipid binding has been associated with fungal virulence and modulation of host immune responses, the structural similarity between Mp1p-LBD2 and HsbAs prompted us to investigate the potential fatty acid-binding properties of HsbA proteins. AlphaFold3 predicts that HsbA1, HsbA2, and HsbA3 adopt highly similar five-helix architectures, implying a shared structural basis for function. This fold suggests potential interactions with hydrophobic molecules, supported by docking and co-folding analyses showing that fatty acids such as palmitic and arachidonic acid fit within the cavity. Although both palmitic acid (PA) and arachidonic acid (AA) were predicted to bind within the conserved hydrophobic cavity of HsbA proteins, the different binding preferences observed among HsbA1–HsbA3 indicate a degree of ligand specificity. These differences can be explained by the distinct structural properties of the two fatty acids. Arachidonic acid, a 20-carbon polyunsaturated fatty acid containing four *cis* double bonds, possesses considerable conformational flexibility, which may facilitate its accommodation within the wider and more flexible binding cavity of HsbA3. In contrast, the fully saturated, linear 16-carbon chain of palmitic acid is likely better suited to the narrower binding channel of HsbA1. These findings suggest that HsbA proteins recognize long-chain fatty acids primarily through hydrophobic interactions while retaining ligand-specific binding preferences that are influenced by the architecture of the binding pocket. Nevertheless, these predictions should be interpreted cautiously, as computational methods do not fully capture the conformational flexibility of proteins during ligand binding. Consequently, experimental validation will be required to determine whether the predicted differences in fatty acid recognition reflect the physiological functions of HsbA proteins. The most informative insights come from the interfacial behavior of these proteins. Molecular dynamics simulations show that, despite being globally hydrophilic, HsbA proteins reorient toward hydrophobic phases (Fan et al., 2006). A localized hydrophobic patch, mainly at the C terminus, involving the loop connecting helices 3 and 4, acts as an anchor that tethers the protein to hydrophobic surfaces (Hakanpää et al., 2006). This behavior varies within the family: HsbA1 and HsbA2 remain mostly in the aqueous phase while maintaining stable interfacial contact, whereas HsbA3 forms stronger and more persistent interactions with the hydrophobic phase. These computational findings align with experiments: the increased surface hydrophobicity in *hsbA1–3* overexpression strains, along with enhanced biofilm formation and altered surface morphology, follow from this interface-binding behavior. The stronger hydrophobic interaction predicted for HsbA3 matches its more pronounced phenotypes, including altered growth, stress sensitivity, and spore properties. In addition, the ability of HsbA proteins to trap hydrophobic molecules in their internal cavity offers a mechanism by which surface binding can be stabilized or amplified at the molecular level (Lam et al., 2019). Although the biological consequences of fatty acid binding by *M. lusitanicus* HsbA proteins remain experimentally unconfirmed, this property may influence protein localization, surface association, or interactions with host-derived lipids. The similarity to Mp1p-LBD2, which binds arachidonic acid and contributes to modulation of host inflammatory responses in *Talaromyces marneffei*, raises the possibility that HsbA-mediated lipid interactions could also contribute to host–fungus communication.

All tested *hsbA* genes showed low early expression followed by gene-specific induction, with *hsbA1a*, *hsbA2*, and *hsbA3* peaking at days 3, 4, and 6, respectively. This temporal profile, together with temperature-dependent regulation (*e.g.*, *hsbA2* downregulated and *hsbA3* upregulated at 35 °C), indicates that HsbA proteins are not constitutive structural components but are instead linked to specific developmental and environmental transitions (Cai et al., 2021). Altering individual *hsbA* genes caused compensatory expression changes, such as increased *hsbA2*, implying functional and regulatory connectivity within the family. Growth phenotypes correlate with the timing of *hsbA* induction: disrupting their balance likely interferes with normal developmental progression, consistent with reduced colony expansion upon *hsbA1a* or *hsbA3* overexpression. Temperature further shifts this balance, as the altered *hsbA2* and *hsbA3* expression at 35 °C matches the growth behavior of the corresponding mutants, suggesting these genes contribute to environmental stress adaptation, a process known to be important for the survival and pathogenicity of Mucorales (Singh et al., 2016). The compensatory behavior of the genes when one is altered indicates a connected system rather than independent functions.

Under cell wall–targeting stress (*e.g.*, after Congo Red and Calcofluor White treatment), most strains became more resistant when *hsbA* genes were deleted or overexpressed, except for the *hsbA1* overexpression strain and the *hsbA2* disruption mutant. This implies that altered HsbA levels trigger compensatory mechanisms that enhance cell wall integrity. Under SDS-induced membrane stress, effects were gene-specific: *hsbA2* disruption and *hsbA3* overexpression reduced growth. Thus, HsbA proteins support cell wall stability, with *hsbA2* likely promoting membrane resilience and elevated *hsbA3* disrupting normal processes. Reduced growth of the *hsbA3* overexpression strain may also result from delayed germination, linking stress sensitivity to developmental timing.

A central result is that HsbA-dependent surface remodeling is linked to development (Bayry et al., 2012). Overexpression strains lost the typical spiny sporangial outer layer, whereas *hsbA2* disruption caused extracellular matrix–like accumulation on the hyphal surface. Similar ECM production has been described in aerial colonies of *Aspergillus fumigatus*, where the matrix contributes to biofilm organization (Beauvais et al., 2007). These structural changes coincided with altered biofilm architecture (Mitchell et al., 2016). Because fungal biofilms rely on an extracellular matrix that provides structural cohesion and surface adhesion (Mitchell et al., 2016), the altered extracellular material observed in the *hsbA2* disruption strain may contribute to the changes in biofilm organization. Contact angle measurements confirmed that HsbA proteins strongly modulate surface hydrophobicity, increasing it in overexpression strains and sharply reducing it after *hsbA2* disruption. In filamentous fungi, surface hydrophobicity is typically established during sporulation through the deposition of surface-active proteins on spores and aerial structures (Cai et al., 2021). Consistent with this general pattern, our data suggest that the effects of HsbA proteins on colony hydrophobicity are largely associated with spore formation and spore hydrophobicity.

Phagocytosis assays showed increased uptake of spores from HsbA overexpression strains by J774.2 macrophages, indicating enhanced initial host–pathogen contact, likely due to altered surface architecture and increased hydrophobicity. HsbA-dependent regulation also affects germination timing and progression, shifting the spores’ developmental state during host interaction. Thus, differences in macrophage survival likely result from exposure to different developmental stages with distinct susceptibilities, rather than intrinsic changes in killing resistance. LDH data showed greater host cell damage in both cell lines following interaction with the two overexpression strains (MS12+pAV1 and MS12+pAV2), likely reflecting longer interaction times and altered host–pathogen dynamics rather than stronger early fungal killing (Westman et al., 2019). This timing-based explanation (Lee et al., 2015) also helps to reconcile the *in vitro* and *in vivo* results: in both insect hosts, *hsbA* disruption increased virulence, whereas overexpression reduced pathogenicity. Differences in virulence likely stem from how host cells respond after contact (Cottier and Pavelka, 2012). We therefore examined IL-8, a key chemokine in early innate immunity that recruits leukocytes (Das et al., 2010). Overexpression of *hsbA1a* and *hsbA2* significantly reduced IL-8 production, with *hsbA3* showing a similar but non-significant trend, indicating dampened early inflammatory signaling due to altered fungal surface properties (Aimanianda et al., 2009). HsbA-driven surface remodeling may weaken early immune response, allowing prolonged fungal persistence at the host interface and translating reduced inflammation into increased host cell damage over time (Montaño and Voigt, 2020). LDH assays supported this, showing greater host cell damage in *hsbA1a* and *hsbA2* overexpression strains, linking reduced inflammatory signaling to increased cytotoxicity during host–fungal interaction. This uncoupling of cytotoxicity from immune activation suggests that HsbA-mediated surface remodeling alters both the physical interaction with host cells and the quality of the immune response. Increased host cell damage with reduced cytokine signaling likely reflects prolonged or altered host contact rather than higher intrinsic fungal toxicity (Westman et al., 2019). These findings indicate that HsbA proteins are not classical virulence factors (Wösten, 2001) but primarily affect developmental timing (*e.g.*, sporangiogenesis, sporulation and germination) and surface properties (including hydrophobicity, sporangial architecture, and biofilm formation), indirectly shaping infection efficiency. Because of these functions, HsbA proteins indirectly control how the fungus interacts with its environment and with host immune cells.

Across filamentous fungi, surface-associated proteins act at the fungus–environment interface by modulating masking, molecular interactions, or substrate attachment. RodA in *A. fumigatus* forms a hydrophobic layer that masks immunogenic cell wall components (Bayry et al., 2012); Mp1p in *T. marneffei* binds host lipid mediators to dampen inflammation (Sze et al., 2017); and HsbA in *A. oryzae* recruits extracellular enzymes to hydrophobic substrates, promoting degradation and nutrient uptake (Ohtaki et al., 2006). These proteins function as interface regulators that convert surface properties into biological outcomes affecting survival, interaction, and adaptation. However, their outputs differ: RodA is mainly a structural immune-evasion layer (Aimanianda et al., 2009), Mp1p a biochemical effector of host signaling (Sze et al., 2017), and *A. oryzae* HsbA an enzymatic recruitment scaffold (Ohtaki et al., 2006). HsbA proteins in *M. lusitanicus* further expand this spectrum by linking surface remodeling to developmental timing. Unlike hydrophobins such as RodA, our data do not support a model in which HsbA proteins form a continuous protective layer covering the spore surface. Instead, our results suggest that HsbA proteins act as secreted surface-associated modulators that influence fungal surface properties, including hydrophobicity, sporangial architecture, and biofilm formation. Whether HsbA proteins are distributed uniformly across the spore surface or localized to specific hydrophobic regions remains to be experimentally determined. *Mucor* HsbA proteins couple changes in surface hydrophobicity or architecture to the transition from dormancy to growth, thereby modulating exposure to host immunity (Chibucos et al., 2016). Mucorales fungi can evade host immunity and rapidly shift from dormant spores to invasive hyphae, a key step in infection (Ibrahim et al., 2012). Because germination is a critical bottleneck in this transition (Sephton-Clark et al., 2018), clarifying how *hsbA* genes affect spore formation and early development is essential for understanding their role in virulence. Our data show that HsbA proteins are not broadly required for asexual sporulation (Wösten, 2001; Nicolás et al., 2008), as most mutants produced spores at levels comparable to the control strain, although *hsbA3* overexpression increased sporulation efficiency, revealing a gene-specific effect. HsbA proteins strongly affected spore morphology: disruption of *hsbA2* or *hsbA3* increased spore size and raised the proportion of enlarged spores. Rather than directly determining spore dimensions, HsbA proteins may influence spore size and germination indirectly through effects on spore surface maturation and wall organization. This interpretation is supported by our SEM and ConA-FITC analyses (**Figures 7A–E**): HsbA overexpression strains showed loss of the characteristic external spiny sporangial layer, whereas the *hsbA2* disruption mutant exhibited ECM-like material accumulation on the hyphal surface and altered accessibility of mannose- and glucose-containing extracellular polysaccharides. These findings suggest that HsbA-dependent regulation of surface properties contributes to proper spore maturation and developmental progression. Similar relationships between fungal surface properties and development have been described for hydrophobins, which influence aerial structure formation, spore surface characteristics, and maturation processes (Aimanianda et al., 2009; Bayry et al., 2012; Valsecchi et al., 2018; Cai et al., 2021). These structural changes had direct functional consequences for germination and infection. In Mucorales, spore size and germination capacity are often correlated with virulence (Li et al., 2011), and reduced spore size has previously been associated with altered growth dynamics and infection potential. However, as reported in other systems, reduced spore size does not universally translate into lower virulence, suggesting that compensatory mechanisms may modulate infection outcomes depending on the genetic background (Lax et al., 2020).

Although *hsbA3* overexpression resulted in the most evident sporulation phenotype, our data indicate that HsbA1, HsbA2, and HsbA3 do not have fully redundant functions. The effects of *hsbA1* and *hsbA2* manipulation were mainly associated with changes in surface properties, including hydrophobicity, biofilm formation, and extracellular matrix organization. Notably, altered *hsbA1* expression was consistently accompanied by increased *hsbA2* transcript levels, suggesting that these two genes may be functionally connected and that *hsbA2* induction could partially compensate for changes in *hsbA1* expression. In contrast, *hsbA3* showed a more distinct expression profile, and its overexpression increased sporulation capacity and affected sporangial morphology, indicating a specific contribution to sporangiogenesis. These findings suggest that, despite their similarity, individual HsbA proteins may have specialized roles during fungal surface remodeling and development.

## Conclusion

**Figure 11:**
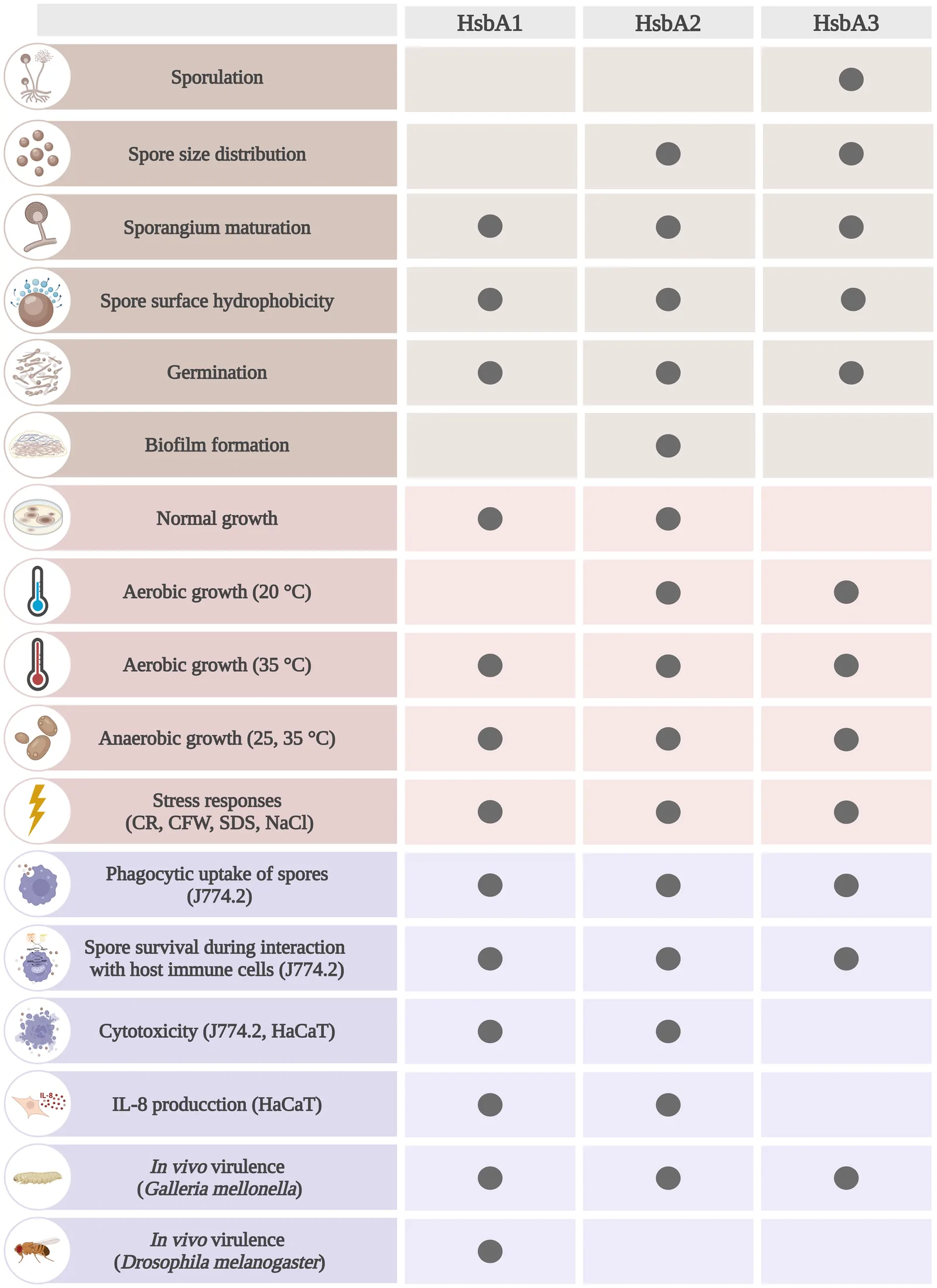
Summary of the phenotypic and virulence-associated functions of HsbA1, HsbA2, and HsbA3 in *Mucor lusitanicus*. The figure illustrates the involvement of the individual HsbA proteins in developmental processes. In addition, the figure summarizes their roles in host–pathogen interactions, including phagocytic uptake, survival during interaction with immune cells, cytotoxicity, IL-8 induction, and *in vivo* virulence in *Galleria mellonella* and *Drosophila melanogaster* infection models. Black circles indicate experimentally observed involvement of the given HsbA protein in the corresponding phenotype or biological process. Created in BioRender. https://BioRender.com/ujuyhgo.

The three HsbA proteins have overlapping roles, but each one also shows a main specific function during the development and host interaction of *M. lusitanicus* (**Figure 11**). HsbA1 is the key player in virulence and host interaction, having the strongest impact on pathogenicity *in vivo*. HsbA2 is mostly connected to biofilm formation, pointing to a more specific role in surface-associated growth and environmental persistence. HsbA3 is primarily involved in sporulation, influencing several stages of sporangiogenesis, including spore germination, viability, and overall developmental progression. All three proteins still contribute to stress adaptation and virulence. Changing *hsbA* expression consistently affected virulence *in vivo*: gene disruption generally increased pathogenicity, while overexpression reduced it. Hydrophobic-binding proteins are important mediators of surface interactions and adhesion, crucial for biofilm formation and environmental persistence. In *M. lusitanicus*, HsbA proteins seem to extend this role beyond adhesion, integrating surface properties with developmental timing and host interaction dynamics, thereby shaping environmental adaptation and infection outcomes.

## Materials and methods

### Sequence analysis of HsbA proteins

HsbA proteins of *Mucor lusitanicus* were identified by Pfam domain (HsbA, PF12296) (Ohtaki et al., 2006; Muszewska et al., 2017) annotations in the *Mucor lusitanicus* genome database (DoE Joint Genome Institute; *Mucor lusitanicus* CBS277.49 v3.0; https://mycocosm.jgi.doe.gov/Mucci3/Mucci3.home.html). This search identified eight putative HsbA homologs, designated as HsbA1a (Protein ID: 1482774), HsbA1b (1482775), HsbA2 (1442756), HsbA3 (1474024), HsbA4 (1432553), HsbA5 (1594666), HsbA6 (1522749) and HsbA7 (1567658). *M. lusitanicus* HsbA proteins’ length, molecular weight and genomic location were retrieved from the genome database. Signal sequence features and signal sequence cleavage sites were identified using SMART (Simple Modular Architecture Research Tool) (https://smart.embl.de/) (Letunic and Bork, 2026). Pairwise sequence similarity analyses were conducted using Clustal Omega (https://www.ebi.ac.uk/jdispatcher/msa/clustalo) (Madeira et al., 2024) including amino acid sequence similarity analysis of HsbA proteins, and nucleotide sequence alignment of *hsbA1a* and *hsbA1b* coding sequences with their promoter and terminator regions. For the pairwise comparison of *hsbA1a* and *hsbA1b*, 500 bp upstream (promoter region) and downstream (terminator region) of the coding sequences were included in the analysis. To assess the relative phylogenetic position of proteins showing >30% sequence similarity with *M. lusitanicus* HsbA proteins, sequence alignment was performed using Clustal Omega (https://www.ebi.ac.uk/jdispatcher/msa/clustalo) (Madeira et al., 2024). The dendrogram generated from the sequence alignment was exported and visualized using iTOL v7 (Letunic and Bork, 2024), with branch lengths indicated.

### *In silico* molecular modelling

Hydrophobic and hydrophilic surface patches of the final molecular dynamics conformations were visualized using UCSF ChimeraX (Pettersen et al., 2021) to generate the representations shown in **Supplementary Figure 3**. Alphafold 3 (Abramson et al., 2024), the latest version of artificial intelligence-based protein structure prediction algorithm was used to predict structures for all three proteins while noting the confidence scores (ipTM) in each case. Autodock Vina (Eberhardt et al., 2021) was used for all docking experiments with palmitic acid (PA) and arachidonic acid (AA) on the grid size defined based on crystal structure of Mp1p protein bound to palmitic acid (Pdb ID: 5E7X) (Liao et al., 2010). Further, co-folding of protein and ligand structures was also carried out using AI-based Boltz-1 (Wohlwend et al., 2025) and Boltz-2 (Passaro et al., 2025) to compare the results with empirical methods like Vina. For molecular dynamics simulations of the folded proteins, HsbA1, HsbA2 and HsbA3, were placed at the interface of water and hexane layers to ascertain their mode of binding. Packmol (Martínez et al., 2009) was used to prepare the systems and AmberTools (Case et al., 2023) ‘tleap’ was used to generate the topology and coordinate files. Both systems were prepared for the classical simulation run in 5 consecutive steps, starting with minimization of the solvent atoms for 10, 000 steps followed by heating of the solvent for 1 ns. The next steps include running 3 consecutive equilibration steps with an NPT ensemble with the last step without any restraints on the system. The production run was carried out for 50 ns without restraints. The Amber22 (Case et al., 2022) module available at the Komondor supercomputer via the KIFU facility was utilized for all calculations. The complex formation was predicted using Alphafold multimer accessed via Google’s Colabfold and their respective binding affinity was calculated using the Prodigy (Vangone and Bonvin, 2015; Xue et al., 2016) (https://rascar.science.uu.nl/prodigy/) web server.

### Strains, media, and growth conditions

The *Mucor lusitanicus* MS12 strain, a leucine- and uracil-auxotrophic (*leuA^−^*, *pyrG^−^*) derivative of the wild-type *M. lusitanicus* CBS277.49 (Benito et al., 1992), was used for mutant generation. The MS12+*pyrG* strain, complemented with a functional *pyrG* allele (Ibragimova et al., 2020), served as a control in all phenotypic analyses. During this study, the following genetically modified strains were generated and used: the gene disruption mutants MS12-*ΔhsbA1a*+*pyrG*, MS12-*ΔhsbA2*+*pyrG*, and MS12-*ΔhsbA3*+*pyrG*, as well as the overexpression strains MS12+pAV1, MS12+pAV2, and MS12+pAV3, carrying overexpression constructs for *hsbA1a*, *hsbA2*, and *hsbA3*, respectively. All strains were derived from the MS12 background and employed in the phenotypic and functional analyses described in this study. Fungal strains were routinely maintained on solid yeast nitrogen base medium supplemented with leucine (YNB+L; 10 g L^−1^ D-glucose, 1.5 g L^−1^ (NH_4_)_2_SO_4_, 1.5 g L^−1^ Na-L-glutamate, 0.5 g L^−1^ YNB; supplemented with distilled water up to 500 mL, pH 4.5; when required, 0.5 g L^−1^ leucine was added for selection; for solid medium, 20 g L^−1^ agar was added) at 25 °C until use. Mitotic stability of transformants was assessed on malt extract agar (MEA; 10 g L^−1^ glucose, 5 g L^−1^ yeast extract, 10 g L^−1^ malt extract, and 20 g L^−1^ agar), a non-selective complete medium. For experimental assays, sporangiospores were collected from plate-grown cultures on MEA, or YNB+L media, depending on the experimental setup, and used for inoculation. Prior to polyethylene glycol (PEG)-mediated transformation (Benito et al., 1992), cultures were grown on yeast extract - glucose medium (YEG; 10 g L^−1^ glucose, 5 g L^−1^ yeast extract; supplemented with distilled water up to 500 mL; for solid medium, 20 g L^−1^ agar was added) at 25 °C for 24 h. Growth experiments were performed on solid YNB+L; to assess temperature-dependent growth, 10^4^ spores were point-inoculated onto agar plates and incubated at 20 °C, 25 °C, or 35 °C. For stress sensitivity assays, spores were plated on YNB+L medium supplemented with Congo Red (CR; 2 mg mL^−1^) (Acros Organics, Geel, Belgium), Calcofluor White (CFW; 0.1 mg mL^−1^) (Sigma-Aldrich, St. Louis, MO, USA), sodium dodecyl sulfate (SDS; 0.004%, w/v), or NaCl (0.8 M). Colony diameters were measured and expressed relative to control conditions. Anaerobic growth was performed using a BBL GasPak system (Becton Dickinson, Franklin Lakes, NJ, USA) with Anaerocult C (Merck, Darmstadt, Germany). Plates were incubated at 20 °C, 25 °C, or 35 °C for 4 days prior to analysis. For nucleic acid extraction, 10^6^ sporangiospores were inoculated onto YNB+L plates and incubated at 25 °C or 35 °C for 4 days. Murine macrophage-like J774.2 cells (Invitrogen, Thermo Fisher Scientific, Waltham, MA, USA) and human keratinocyte HaCaT (Boukamp et al., 1988; Deyrieux and Wilson, 2007) cells were used for *in vitro* interaction assays. Cells were cultured at 37 °C in 5% CO_2_ in Dulbecco’s Modified Eagle Medium (DMEM; high glucose, 4.5 g L^−1^; Capricorn Scientific, Ebsdorfergrund, Germany, Lot No: CP41-13-09) supplemented with 10% heat-inactivated fetal bovine serum (FBS) (Capricorn Scientific, Ebsdorfergrund, Germany), penicillin (100 U mL^−1^) (Lonza Group, Basel, Switzerland), and streptomycin (100 μg mL^−1^) (Lonza Group, Basel, Switzerland). Cells were maintained at a density of 1 × 10^5^ cells mL^−1^, and the DMEM medium was replaced every 2 days. *In vivo* pathogenicity assays were performed using *Galleria mellonella* larvae and *Drosophila melanogaster* Oregon R strains, kindly provided by the research group of Rita Sinka (Department of Genetics, University of Szeged, Hungary). *Escherichia coli* DH5α was used as a host strain and transformed using the Mix & Go™ kit (Zymo Research, Irvine, CA, USA). Bacterial cultures were grown in Luria–Bertani (LB; 10 g L^−1^ tryptone, 5 g L^−1^ yeast extract, 10 g L^−1^ NaCl; pH adjusted to 7.0; for solid medium, 20 g L^−1^ agar was added; for selection of *E. coli* transformants, ampicillin (Amp, AppliChem, Darmstadt, Germany) was added to a final concentration of 50 µg mL^−1^ to the medium and incubated at 37 °C until experimental use.

### Molecular techniques

Genomic DNA and total RNA were extracted using the ZR Fungal/Bacterial DNA MiniPrep (Zymo Research, Irvine, CA, USA) and Direct-zol RNA MiniPrep (Zymo Research, Irvine, CA, USA) kits, respectively, following the manufacturer’s protocols. To amplify genes or gene fragments from genomic DNA, Phusion High Fidelity DNA Polymerase (Thermo Scientific, Waltham, MA, USA) was employed according to the manufacturer’s recommendations. The resulting PCR products were purified and concentrated using the Zymoclean Large Fragment DNA Recovery Kit (Zymo Research, Irvine, CA, USA) and the DNA Clean & Concentrator-5 (Zymo Research, Irvine, CA, USA). Reverse transcription was performed using the Maxima H Minus First Strand cDNA Synthesis Kit (Thermo Fisher Scientific, Waltham, MA, USA) with random hexamer and oligo(dT)_18_ primers, in accordance with the manufacturer’s instructions. Quantitative real-time PCR (qRT-PCR) analysis was conducted on a CFX96 real-time PCR detection system (Bio-Rad, Hercules, CA, USA), using the Maxima SYBR Green qPCR Master Mix (Thermo Fisher Scientific, Waltham, MA, USA) and primers listed in **Supplementary Table 1**. Relative quantification of gene expression and copy number was determined using the threshold cycle (2^−ΔΔCT^) method (Livak and Schmittgen, 2001), with the *M. lusitanicus* actin gene serving as the reference (Dankai et al., 2015). The amplification protocol consisted of an initial denaturation at 95 °C for 3 minutes, followed by 40 cycles of 95 °C for 15 seconds, 60 °C for 30 seconds, and 72 °C for 30 seconds. All experiments were conducted in both biological and technical triplicates.

### Disruption of the *hsbA* genes using the CRISPR-Cas9 method

To achieve targeted disruption of *hsbA1a*, *hsbA2*, and *hsbA3* in *Mucor lusitanicus*, CRISPR–Cas9-mediated genome editing was employed, and its plasmid-free version was selected to minimize *off-target* effects (Pattanayak et al., 2013), as described previously (Nagy et al., 2017; Ibragimova et al., 2020). Protospacer sequences were designed to direct Cas9 cleavage within the coding regions of each gene. The selected 20-nucleotide protospacers were 5′-ACTGACTGCTGTGCAGTCGC-3′ (*hsbA1a*), 5′-GAAACGCGTCTCAAGAAAGC-3′ (*hsbA2*), and 5′-GAATTTGATGCCGTTCTTT-3′ (*hsbA3*), corresponding to regions located 310–330 bp, 282–302 bp, and 426–446 bp downstream of the respective start codons. Based on these sequences, Alt-R CRISPR crRNA and tracrRNA molecules were designed and synthesized by Integrated DNA Technologies (IDT, Coralville, IA, USA). crRNA:tracrRNA duplexes were assembled in Nuclease-Free Duplex Buffer according to the manufacturer’s instructions. Gene disruption was achieved via homology-directed repair (HDR) following the strategy described by Nagy et al. (2017). Disruption cassettes were generated as described above and used as HDR templates. Each cassette contained approximately 1.1–1.2 kb homologous regions flanking the Cas9 target site, fused to the *M. lusitanicus pyrG* selection marker (CBS277.49v2.0 genome database ID: Mucci1.e_gw1.3.865.1), including its native promoter and terminator. For *hsbA1a*, homologous arms of 1109 bp (upstream) and 1129 bp (downstream) were amplified and fused with *pyrG* using nested primers McHsbA1a/7 and McHsbA1a/8 (**Supplementary Table 1**), with fragments combined in equimolar ratios (1:1:1) during fusion PCR. A similar approach was used for *hsbA2* (1108 bp and 1138 bp arms; primers McHsbA2/7 and McHsbA2/8) and *hsbA3* (1155 bp and 1106 bp arms; primers McHsbA3/7 and McHsbA3/8). Transformation was performed by PEG-mediated protoplast transformation (Benito et al., 1992) of the MS12 strain, co-delivering 5 μg HDR template DNA, 10 μM gene-specific guide RNA, and 10 μM Cas9 nuclease (Alt-R^®^ S.p. Cas9 Nuclease; IDT, Coralville, IA, USA). Transformants were selected on solid YNB+L medium via uracil auxotrophy complementation mediated by the *pyrG* marker. Monosporangial isolates were obtained from primary transformants to ensure genetic homogeneity. Transformation efficiencies differed among targets, yielding 8, 2, and 14 transformants per 10^5^ protoplasts for MS12-*ΔhsbA1a*+*pyrG*, MS12-*ΔhsbA2*+*pyrG*, and MS12-*ΔhsbA3*+*pyrG*, respectively. Integration of the disruption cassette at the expected genomic loci was confirmed by PCR using primers listed in **Supplementary Table 1**, producing fragments of the anticipated sizes (**Supplementary Figure 4A**). From each transformation, two independent isolates were selected for further analysis. The resulting mutant strains exhibited mitotic stability on both MEA and YNB+L media, retaining the integrated construct after 20 cultivation cycles. Successful gene inactivation was confirmed by quantitative RT-PCR, which demonstrated the absence of *hsbA1a*–*3* transcripts in all validated transformants (**Supplementary Figure 4B**).

### Overexpression of *hsbA1a*, *hsbA2*, and *hsbA3* genes of *Mucor lusitanicus*

For the overexpression of the *hsbA1a*, *hsbA2*, and *hsbA3* genes, plasmids pAV1, pAV2, and pAV3 were constructed, respectively. The coding sequences of the target genes were amplified from *M. lusitanicus* genomic DNA using specific primer pairs: McHsbA1oefw/McHsbA1oerev for *hsbA1a*, McHsbA2oefw/McHsbA2oerev for *hsbA2*, and McHsbA3oefw/McHsbA3oerev for *hsbA3* (primer sequences listed in **Supplementary Table 1**). The resulting PCR products were cloned between the promoter and terminator regions of the *M. lusitanicus* glyceraldehyde-3-phosphate dehydrogenase 1 (*gpd1*) gene (EMBL Accession No.: AJ293012) within the pPT81 expression vector (Csernetics et al., 2011; Nagy et al., 2014). The pPT81 vector also contained the *M. lusitanicus pyrG* gene (CBS277.49v2.0 genome database ID: Mucci1.e_gw1.3.865.1), which encodes orotidine-5’-monophosphate decarboxylase (*pyrG*) and serves as a selectable marker by complementing the uracil auxotrophy of the MS12 strain. Transformation frequencies were determined to be 11, 2, and 7 transformants per 10^5^ protoplasts for MS12+pAV1, MS12+pAV2, and MS12+pAV3, respectively. The presence of the plasmids in the transformants was verified by PCR amplification (**Supplementary Figure 4C**), yielding the expected fragment sizes of 750 bp for pAV1 and pAV2 and 752 bp for pAV3, and sequencing of the amplified products confirmed their identity. Overexpression of the target genes in the selected transformants was confirmed by quantitative RT-PCR, demonstrating significantly elevated transcript levels compared to the control strain (**Supplementary Figure 4D**). From the transformation experiment two isolates from each strain were selected for the further experiments.

### Whole genome sequencing and analysis

To further exclude unintended genomic alterations, whole-genome sequencing (WGS) (Smith et al., 2014) was performed on two randomly selected mutants (MS12-*ΔhsbA1a*+*pyrG* and MS12-*ΔhsbA2*+*pyrG*). The analysed samples included the parental MS12 strain as well as the mutant strains MS12-*ΔhsbA1a*+*pyrG* and MS12-*ΔhsbA2*+*pyrG*. WGS sequencing libraries were generated from sonicated genomic DNA with NEBNext Ultra II DNA Library Prep Kit for Illumina (New England Biolabs, Ipswich, MA, USA) and sequenced using an Illumina MiSeq DNA sequencing instrument with MiSeq Reagent kit v3-600 (Illumina, San Diego, CA, USA). WGS was conducted Raw sequencing reads were aligned to the reference genome (*Mucor lusitanicus*, assembly: Mucor_lusitanicus_v2_masked_scaffolds.fasta, available at the JGI portal) following rigorous quality control procedures. The sequencing data is available in the NCBI SRA database (NCBI SRA database ID: PRJNA1463750). Initial quality assessment of FASTQ files was performed using FastQC (Babraham Bioinformatics; https://www.bioinformatics.babraham.ac.uk/projects/fastqc/) and TrimGalore (RRID: SCR_011847), the latter of which also trimmed adapter sequences and discarded reads shorter than 36 bp post-trimming. High-quality paired-end reads were then mapped to the reference genome using the BWA-MEM algorithm (Li and Durbin, 2009; Li, 2013). Mapping quality and coverage were visualized via Qualimap (Okonechnikov et al., 2016). Prior to variant calling, PCR duplicates and technical replicates were identified and marked using GATK/Picard’s MarkDuplicates (Picard, 2019; https://broadinstitute.github.io/picard/) and excluded from downstream analyses through SAMtools flagging. Variant detection was carried out employing multiple tools—SAMtools (Li et al., 2009)/bcftools (Li, 2013), FreeBayes (RRID: SCR_010761), and Bedops v2.4.39 (Neph et al., 2012) —to ensure comprehensive identification of genomic alterations. Validation of targeted gene disruptions was performed by interrogating BAM files for coverage at the edited loci using BLAST searches (Camacho et al., 2009). Regions lacking read coverage in mutant samples, relative to the reference, were deemed successfully disrupted. Variant analysis comparing these strains to the parental MS12 genome revealed the expected targeted disruptions without detectable *off*-*target* mutations (Pattanayak et al., 2013), insertions, or deletions (**Supplementary Table 3**). Analysis of single nucleotide polymorphisms (SNPs) and indels showed no significant differences relative to the parental strain (**Supplementary Table 3**).

### Germination assay

Sporangiospores were harvested from 7-day-old cultures grown on YNB+L and incubated at 25 °C. To synchronize the physiological state of the spores, they were transferred to liquid YNB+L medium and incubated at 4 °C for 16 hours. For the germination assay, 10^7^ spores were inoculated into 10 mL of liquid YNB+L and incubated at 25 °C under continuous shaking at 150 rpm. Germination was monitored by light microscopy (Leica, Wetzlar, Germany) at 8 hours post-inoculation. At each time point, 10 μl of the spore suspension was placed in a Bürker counting chamber (Marienfeld, Lauda-Königshofen, Germany) and germinated spores were counted in 10 randomly selected microscopic fields. The percentage of germination was calculated based on the total number of observed spores.

### Sporulation capacity assay

To assess sporulation efficiency, 10^4^ sporangiospores were point-inoculated onto MEA plates and incubated at 25 °C for four days. After incubation, spores were harvested using sterile 1× phosphate-buffered saline (PBS; Gibco, Thermo Fisher Scientific, Waltham, MA, USA; 137 mM NaCl, 2.7 mM KCl, 10 mM Na_2_HPO_4_, 2 mM KH_2_PO_4_, pH 7.4), followed by centrifugation at 2, 000 × g for 10 minutes at 4 °C. Spore concentrations were quantified using a Bürker counting chamber (Marienfeld, Lauda-Königshofen, Germany), and values were compared between mutant and control strains to determine relative sporulation capacity. The spore concentration was expressed as the number of spores per 1 cm².

### Measurement of the spore sizes

Fungal strains were cultivated on MEA at 25 °C for four days to allow spore maturation. The spores were then stained with 5 mM CFW (Sigma-Aldrich, St. Louis, MO, USA) for 20 minutes under continuous agitation at 100 rpm to enhance fluorescence. After staining, samples were washed three times with 1 mL of 1 x PBS. For analysis, collected samples were centrifuged at 6, 800 × g for 10 min and resuspended in 200 μL 1 x PBS. Samples were measured with a FlowSight imaging flow cytometer (Amnis ImageStream X Mk II imaging flow cytometer; Amnis, Austin, TX, USA), and the associated IDEAS 6.2 software (Dominical et al., 2017) was used for evaluation. Spore size distribution was quantitatively assessed using flow cytometry with a 405 nm excitation laser, acquiring data from 10, 000 events per sample. Spore area was calculated based on forward scatter parameters, and size distribution histograms were generated to categorize spores into small and large populations using a threshold of 125 μm².

### Electron microscopic analysis of the mycelia and sporangiospores

Fungal spores (10^6^/100 µL) were cultivated on YNB+L medium at 25 °C for 7 days. The plates were placed at -80 °C overnight and freeze-dried for 1 day with a Martin Christ Gefriertrocknungsanlagen ALPHA 1–4 freeze dryer. Following drying, the samples were mounted on specimen stubs and sputter-coated with a conductive gold layer using a Quorum Technologies SC 7620 Mini coater. The electron microscope images were acquired at an accelerating voltage of 10 kV using a Hitachi S-4700 Type II FE-SEM (Hitachi High Technologies, USA) (Kedves et al., 2021). For the analysis of the biofilm formation, fungal spores were cultivated on MEA medium at 25 °C for 4 days. The spores were collected with 10 mL of 1 x PBS buffer. Tissue culture coverslips (13 mm; plastic; Sarstedt AG & Co. KG, Nümbrecht, Germany) were placed into the wells of the 24-well plate (SPL Life Science Co, Pocheon, Republic of Korea) followed by the inoculation of spores. The spore concentration was adjusted to 1x10^6^/mL, which incubated in RPMI1640 with L-glutamine and 25 mM HEPES (Capricorn Scientific, Ebsdorfergrund, Germany, Lot No: CP25-8204) medium on a 24-well plate (SPL Life Science Co, Pocheon, Republic of Korea) at 25 °C for 48 hours. The wells were washed with 1 mL of 1 x PBS buffer after the incubation. The fungal biofilm was fixed in 2.5% glutaraldehyde (Sigma-Aldrich, St. Louis, MO, USA) and 0.15% Alcian blue 8GX (Reneal, Budapest, Hungary) containing phosphate buffer (1 X PBS, pH 7.4) for 4 hours. Then the samples were washed two times with 1 mL of 1 x PBS buffer. After post-fixation in 1% OsO4 for 1 hour, the samples were dehydrated in aqueous solutions of increasing ethanol concentrations, critical point dried, covered with 15 nm gold by a Quorum Q150T ES (Quorum Technologies, Lewes, UK) sputter and observed in a JEOL JSM-7100F/LV (JEOL SAS, Croissy-sur-Seine, France) SEM by using 2 kV accelerating voltage. Microscopic analyses were carried out with three biological replicates, each with two technical replicates.

### Fluorescent microscopic analysis of the mycelia

Fungal spores (10^6^/100 µL) were cultivated on YNB+L media at 25 °C for seven days. Mycelial plugs (5 mm in diameter) were excised from the 7-day-old colonies using a sterile cork borer (Sigma-Aldrich, St. Louis, MO, USA) and were placed into the wells of the 24-well plate (SPL Life Science Co, Pocheon, Republic of Korea). The mycelia were stained with 25 μg mL^−1^ Alexa Fluor488 conjugated concanavalin A (Invitrogene, 5 mg; Cat No: C11252) at 25 °C under continuous shaking at 100 rpm for 30 min in the dark. The wells were washed three times with 1 mL of 1× PBS after incubation. The stained mycelia were placed on glass slides (SUPERFROST PLUS; 25 x 75 x 1, 0 mm; Waltham, MA, USA) and covered with microscope cover slips (18x18 mm; VWR International, Radnor, PA, USA). The morphology of the mycelia was examined using an Axiovert 200 M inverted fluorescence microscope (Carl Zeiss, Oberkochen, Germany). ConA-FITC fluorescence was detected at excitation and emission wavelengths of 495 nm and 519 nm, respectively. Images were acquired at 10× magnification, and scale bars represent 100 µm. Microscopic analyses were carried out with three biological replicates, each with two technical replicates.

### MATH assay

To evaluate the hydrophobicity of the spore surfaces, we applied the microbial adhesion to hydrocarbons (MATH) assay, following established protocols with minor modifications (Ramos et al., 2020; Suchodolski et al., 2020). This method quantifies hydrophobicity based on the redistribution of fungal spores between aqueous and hydrocarbon phases after vigorous mixing and phase separation. Cells with higher hydrophobicity preferentially partition into the hydrocarbon phase, leading to reduced optical density in the aqueous layer (Rosenberg, 1984). Fungal cultures were incubated under constant illumination at 25 °C for 4 days. Spores were harvested in sterile distilled water, and suspensions were adjusted to 2 × 10^7^ spores/2 mL. Hexane (500 µl) was added, followed by 1-minute vortexing and 30-minute incubation at 25 °C to allow phase separation. The aqueous phase was carefully collected and the remaining spore concentration was determined using a Bürker chamber (Marienfeld, Lauda-Königshofen, Germany). For each strain, 100 µl of the aqueous phase was transferred to a 96-well plate (8 technical replicates per strain). Absorbance was measured at 600 nm using a SPECTROstar Nano Plate Reader (BMG LABTECH GmbH, Ortenberg, Germany). Controls consisted of spore suspensions without toluene. Hydrophobicity was calculated as a percentage using the formula:


$$Hydrophobicity\;index\;\left(\%\right)=\frac{C_{O}-C_{H}}{C_{O}}\times 100\;$$

where C_O_ is the OD_600_ of the control and CH is the OD_600_ of the aqueous phase. Results represent the mean of three independent biological replicates.

### Contact angle measurement

The fungal colonies were cultured at 25 °C for 4 days by evenly spreading a spore suspension prepared at a concentration of 1 × 10^6^/100 μL onto YNB+L medium covered with cellophane. We placed the mycelium, along with the cellophane, onto a microscopic slide, then added 20 μl of sterile distilled water to the surface of the mycelium. Contact angle measurements of the *hsbA* mutants’ mycelium were performed by using the drop shape analysis system (Easy Drop, Krüss GmbH, Hamburg, Germany). The system was equipped with a steel syringe needle (0.5 mm diameter) and sterile distilled water was used as the test liquid at 25.0 ± 0.5 °C. Using the CCD camera goniometer, the Young – Laplace equation (Montero de Hijes et al., 2020) was used to mathematically characterize the drop contour of the recorded photo using DSA100 software, while at the point of contact of the three-phase contour line slope was defined as the contact angle (Θ). Three biological measurements were conducted with six technical replicates.

### Phagocytosis assay

The murine macrophage-like cell line J774.2 was cultured in DMEM (high glucose, 4.5 g L^−1^; Capricorn Scientific, Ebsdorfergrund, Germany, Lot No: CP41-13-09) supplemented with 10% heat-inactivated FBS (Capricorn Scientific, Ebsdorfergrund, Germany) and 1% 100 × penicillin-streptomycin solution (penicillin (100 U mL^−1^; Lonza Group, Basel, Switzerland); streptomycin (100 μg mL^−1^; Lonza Group, Basel, Switzerland) at 37 °C, 5% CO2 and 100% relative humidity. The murine-like cell line J774.2 (10^4^/100 µl) were seated on a 10-well plate (Greiner Bio-One, Kremsmünster, Austria; cellview cell culture slide, ps, 75/25 mm, glass bottom, 10 compartments, tc, sterile, 5 pcs/tray) 16 h before the experiment. In the phagocytosis assay, the fungal spore suspensions (5 × 10^6^/mL) were stained with 25 µM FUN-1 (Thermo Fisher Scientific, Invitrogen, Waltham, MA, USA) for 30 minutes at 37 °C. After staining, samples were washed three times with 1 mL of 1 x PBS. The collected samples were centrifuged at 6, 800 × g for 5 min and resuspended in 1 mL PBS. Subsequently, the fungal spores (5 x 10^4^/10 µl) were added to the J774.2 macrophages (10^4^/100 µl) at a multiplicity of infection (MOI) of 1:5 (cells:spores). Co-cultures were incubated for 3 hours to facilitate phagocytosis. After the interaction the fungal spores were strained with 10 µM CFW (Sigma-Aldrich, St. Louis, MO, USA) for 15 minutes at 37 °C. The phagocytic index was determined by fluorescence microscope (Axiovert 200 M inverted microscope; Carl Zeiss, Oberkochen, Germany). Chitin in non-phagocytosed spores was detected using 360 nm excitation and 430 nm emission filters, whereas FUN-1 fluorescence in phagocytosed spores was measured with 488 nm excitation and emission collected in the 530 nm (green) and 575–590 nm (red) channels. For each experiment, 10 fields per well were analysed, and at least 1, 000 macrophages per well were quantified using ImageJ software (Schneider et al., 2012). The phagocytic index was the ratio of the number of intracellular fungal spores to the number of macrophages counted. The phagocytosing cells (%) calculated using the following formula:


$$Phagocytosing\;cells\left(\%\right)=\;\frac{the\;number\;of\;phagocytosing\;cells}{the\;total\;number\;of\;macrophage\;cells}\;x\;100$$

and the phagocytosed spores (%) calculated using the following formula:


$$Phagocytosed\;spores\left(\%\right)=\frac{the\;number\;of\;phagocytosed\;spores}{the\;total\;number\;of\;spores}\;x\;100$$

### Assessment of spore viability using the XTT assay

The murine macrophage-like cell line J774.2 (Invitrogen, Thermo Fisher Scientific, Waltham, MA, USA) was cultured in DMEM (high glucose, 4.5 g L^−1^; Capricorn Scientific, Ebsdorfergrund, Germany, Lot No: CP41-13-09) supplemented with 1% heat-inactivated FBS (Capricorn Scientific, Ebsdorfergrund, Germany) and 1% 100 × penicillin-streptomycin solution (penicillin (100 U mL^−1^; Lonza); streptomycin (100 μg mL^−1^; Lonza)) at 37 °C, 100% relative humidity and 5% CO_2_. J774.2 cells (10^4^/100 µl) were seated on a 96-well plate (SPL Life Sciences, Pocheon, Republic of Korea) 16 h before the experiment. For XTT assay spores were collected from strains grown for 4 days at 25 °C. For the assays using pre-treated spores (5 x 10^6^/mL) were incubated for 3 hour in YNB+L medium at 100 rpm to initiate germination, and then spore (5x10^4^/10 µl) added to J774.2 cells (10^4^/100 µl) at a multiplicity of infection (MOI) of 1:5 (cells:spores). In contrast, for the measurements with resting spores, freshly washed spores were used without prior incubation. The experiments were conducted using the Biotium XTT Cell Viability Assay Kit (Biotium Inc., Fremont, CA, USA), following the manufacturer’s instructions. In both cases, the metabolic activity—reflecting cell viability—was monitored over a 3-hour period, with absorbance measurements taken every 1 hour using a SPECTROstar Nano Microplate Reader (BMG LABTECH GmbH, Ortenberg, Germany) at 475 nm (formazan signal) and 650 nm (background). Three independent biological experiments were performed, each with four technical replicates for spores incubated with or without macrophages. During data analysis, background and the macrophage without spores, absorbance was subtracted from all measurements to ensure accuracy. Viability of the spores was calculated using the following formula, where OD_475_ sample was the absorbance measured for the spores incubated with macrophages and OD_475_ control was the absorbance measured for the spores incubated without macrophages:


$$Viability\left(\%\right)=\frac{OD_{475}\;sample}{OD_{475}\;control}\;x\;100$$

### LDH-based cytotoxicity assay of spore-host interactions

The murine macrophage-like cell line J774.2 (Invitrogen, Thermo Fisher Scientific, Waltham, MA, USA) and the HaCaT keratinocytes (MTA-SZTE Dermatological Research Group) (Boukamp et al., 1988; Deyrieux and Wilson, 2007) were cultured in DMEM supplemented with 1% heat-inactivated FBS (Capricorn Scientific, Ebsdorfergrund, Germany) and 1% 100 × penicillin-streptomycin (penicillin (100 U mL^−1^; Lonza Group, Basel, Switzerland); streptomycin (100 μg mL^−1^; Lonza Group, Basel, Switzerland) solution at 37 °C, 5% CO_2_ and 100% relative humidity. J774.2 macrophage-like cells and HaCaT cells (10^5^/mL) were seeded in 24-well plates (SPL Life Sciences, Pocheon, South Korea) and allowed to adhere for 16 h and 24 h, respectively, before the experiment. For the assays, spores harvested from cultures grown for 4 days at 25 °C were collected and incubated (5 × 10^5^/100 µL) with murine macrophage-like J774.2 cells (10^5^/mL) for 16 h and with HaCaT cells (10^5^/mL) for 30 h at a multiplicity of infection (MOI) of 1:5 (cells:spores). During our experiments, we assessed the relative activity of lactate dehydrogenase (LDH) released from the cells using the CyQUANT™ LDH Cytotoxicity Assay Kit (Thermo Fisher Scientific, Invitrogen, Waltham, MA, USA), following the manufacturer’s protocol. After a 16 h and 30 h, respectively, interaction period, supernatant samples were collected and 50 μl aliquots were transferred into 96-well microplates (SPL Life Sciences, Pocheon, South Korea). An equal volume of the reaction mixture was then added to each well, according to the manufacturer’s instructions. Sterile culture medium was used as a negative control, while the supernatant from cells treated with 10% Triton X-100 detergent (Thermo Fisher Scientific, Invitrogen, Waltham, MA, USA) served as a positive control. The plates were incubated for 30 minutes protected from light, after which optical density (OD) readings were taken at 490 nm and 680 nm using a SPECTROstar Nano Microplate Reader (BMG LABTECH GmbH, Ortenberg, Germany), with background subtraction applied. The relative LDH enzyme activity in each sample—used as a measure of cytotoxicity—was calculated using the following formula:

$Cytotoxicity\left(\%\right)=\frac{LDH\;activity\;in\;treated\;sample-spontaneous\;LDH\;activity}{maximum\;LDH\;activity-spontaneous\;LDH\;activity}\;x\;100$

In this context, a value of 100% represents complete cell lysis, whereas 0% indicates intact, undamaged cells.

### Cytokine assay

The HaCaT keratinocytes (MTA-SZTE Dermatological Research Group) (Boukamp et al., 1988; Deyrieux and Wilson, 2007) were cultured in DMEM (high glucose, 4.5 g L^−1^; Capricorn Scientific, Ebsdorfergrund, Germany, Lot No: CP41-13-09) supplemented with 1% heat-inactivated FBS (Capricorn Scientific, Ebsdorfergrund, Germany) and 1% 100 × penicillin-streptomycin solution (penicillin (100 U mL^−1^; Lonza Group, Basel, Switzerland); streptomycin (100 μg mL^−1^; Lonza Group, Basel, Switzerland) at 37 °C, 5% CO_2_ and 100% relative humidity. The HaCaT cells (10^5^/mL) were seeded in 24-well plates (SPL Life Sciences, Pocheon, South Korea) 24 h before the experiment. For the cytokine assay, spores harvested from cultures grown for 4 days at 25 °C were collected and incubated (5 × 10^5^/100 µL) with HaCaT cells (10^5^/mL) for 30 h at a multiplicity of infection (MOI) of 1:5 (cells:spores). To confirm cytokine concentrations in the culture supernatants, DuoSet ELISA kits (R&D Systems, Minneapolis, MN, USA) were used to quantify interleukin (IL)-8 according to the manufacturer’s instructions. Clear Strip-well microplates (Catalog No. DY990, Minneapolis, MN, USA) were used for the assays. As a control, HaCaT cells were incubated in DMEM (high glucose, 4.5 g L^−1^; Capricorn Scientific, Ebsdorfergrund, Germany, Lot No: CP41-13-09) in the absence of fungi. Cytokine titers were calculated by reference to standard curves generated by the four parameters logistic (4PL) curve-fit method (Findlay and Dillard, 2007).

### Survival assay in Drosophila melanogaster

The wild type *Drosophila melanogaster* strain (Oregon-R BL5, Bloomington Drosophila Stock Center) was utilized for all experiments, which were conducted following previously described protocols (Csonka et al., 2021; Jáger et al., 2024). Spore suspensions were prepared in sterile 1 X PBS from 7-day-old cultures grown on YNB+L at 25 °C. Infection was performed by dipping a fine needle into a fungal conidial suspension (10^7^ spore/mL) or sterile PBS for the uninfected control, followed by pricking the thorax of anesthetized flies. Survival was monitored at multiple time points, and flies were transferred to fresh vials every two days. Each experiment was conducted with 45 flies, and the data presented are representative of three independent trials.

### Survival assay in *Galleria mellonella* larvae

Fungal spores were resuspended in insect physiological saline (IPS; 50 mM NaCl, 5 mM KCl, 10 mM EDTA, and 30 mM sodium citrate in 0.1 M Tris-HCl (pH 6.9)) (Bergin et al., 2005). *Galleria mellonella* larvae (Department of Genetics, University of Szeged, Hungary) were inoculated with 10^5^ fungal cells suspended in 20 μL of IPS via injection into the last proleg using 29-gauge insulin needles (BD Micro-Fine, Becton Dickinson, Franklin Lakes, NJ, USA). For each *M. lusitanicus* strain, 20 larvae were infected. Additionally, two control groups were included: an IPS-treated group (uninfected) and an untouched control group (no injections, uninfected), each consisting of 20 larvae. The infected and the control larvae were maintained at 25 °C, and survival was monitored daily for six days. The results presented are representative of at least three independent experiments.

### Statistical analysis

Statistical analyses were performed using GraphPad Prism v8.0.1 software (GraphPad Software, Boston, MA, USA, www.graphpad.com) with a parametric t test. Growth experiments performed under aerobic and anaerobic conditions were analysed using two-way ANOVA. A statistically significant difference was considered when p< 0.05. For *D. melanogaster* and *G. mellonella in vivo* pathogenicity assays, host survival was visualized using GraphPad Prism v8.0.1, applying the Survival Curve function. Significance levels were determined using non-parametric Gehan-Breslow-Wilcoxon and Log-rank (Mantel-Cox) statistical tests.

## Data Availability

The datasets presented in this study can be found in online repositories. The names of the repository/repositories and accession number(s) can be found in the article/**Supplementary Material**.
