# Supplementary material for: Three hydrophobic surface binding a proteins link surface hydrophobicity to sporulation, biofilm formation, and host interaction in Mucor lusitanicus
Source: Front Cell Infect Microbiol. 2026 Aug 20;16:1904531. doi: 10.3389/fcimb.2026.1904531 (PMC13537970; doi:10.3389/fcimb.2026.1904531)
Supplement: Supplementary file 1 [file DataSheet1.pdf]

Supplementary Material

1 Supplementary Figures and Tables

1.1 Supplementary Figures

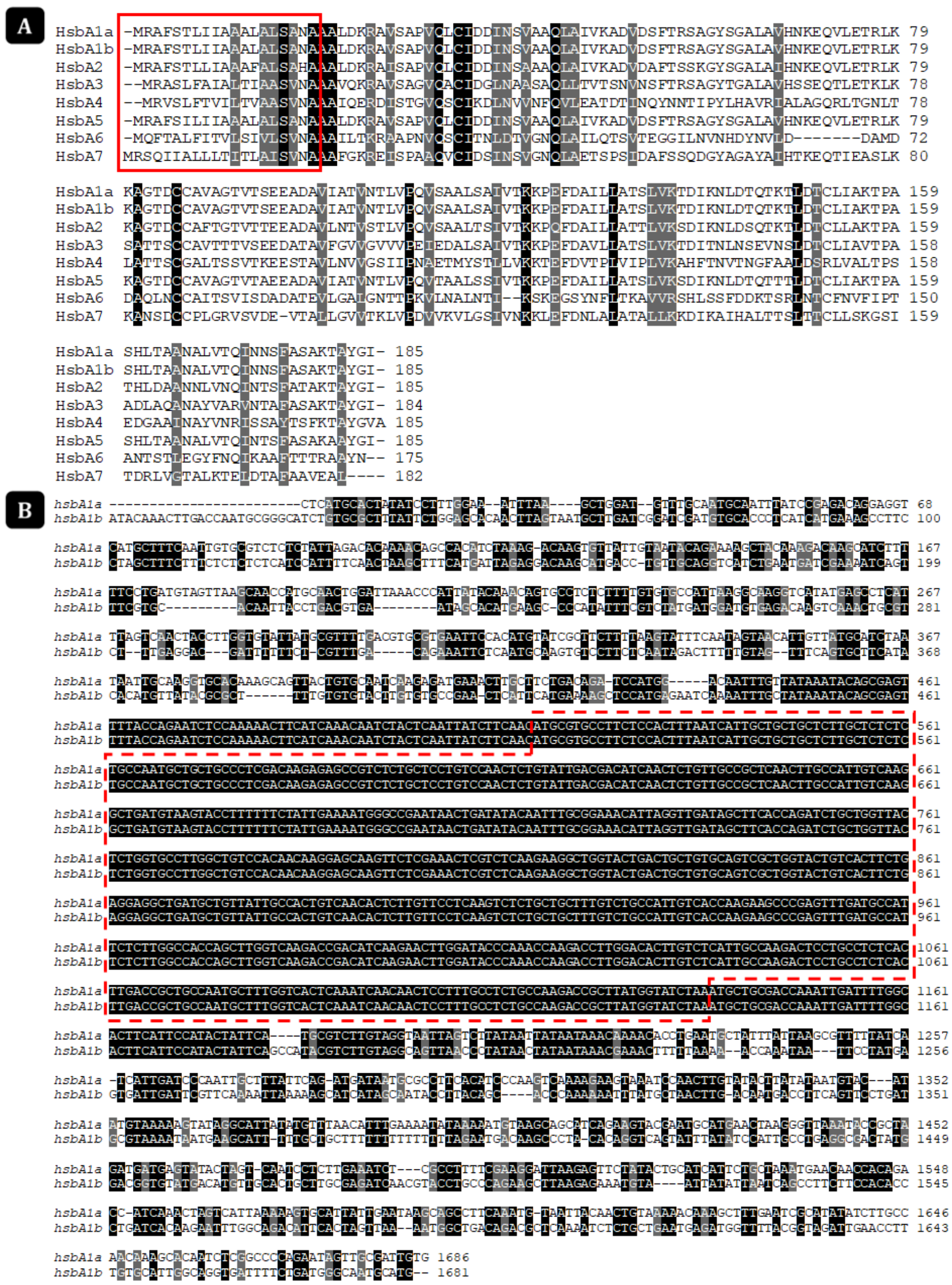

Supplementary Figure 1. (A) Sequence alignment of HsbA1-7 proteins of *Mucor lusitanicus*. Multiple sequence alignment was conducted using Clustal Omega (https://www.ebi.ac.uk/jdispatcher/msa/clustalo) (Madeira et al., 2024). Signal sequences are highlighted in the box in red. Area's shaded in grey represent identical amino acid residues, whereas

those shaded in black represent similar amino acid residues. (**B)** Sequence alignment of HsbA1a and HsbA1b coding sequences with their promoter and terminator regions. The region outlined by the red dashed line indicates the coding region (622 bp); the promoter region is located 500 bp upstream of the coding region, while the terminator region is located 500 bp downstream of the coding region. Sequence alignment was conducted using Clustal Omega (https://www.ebi.ac.uk/jdispatcher/msa/clustalo) (Madeira et al., 2024).

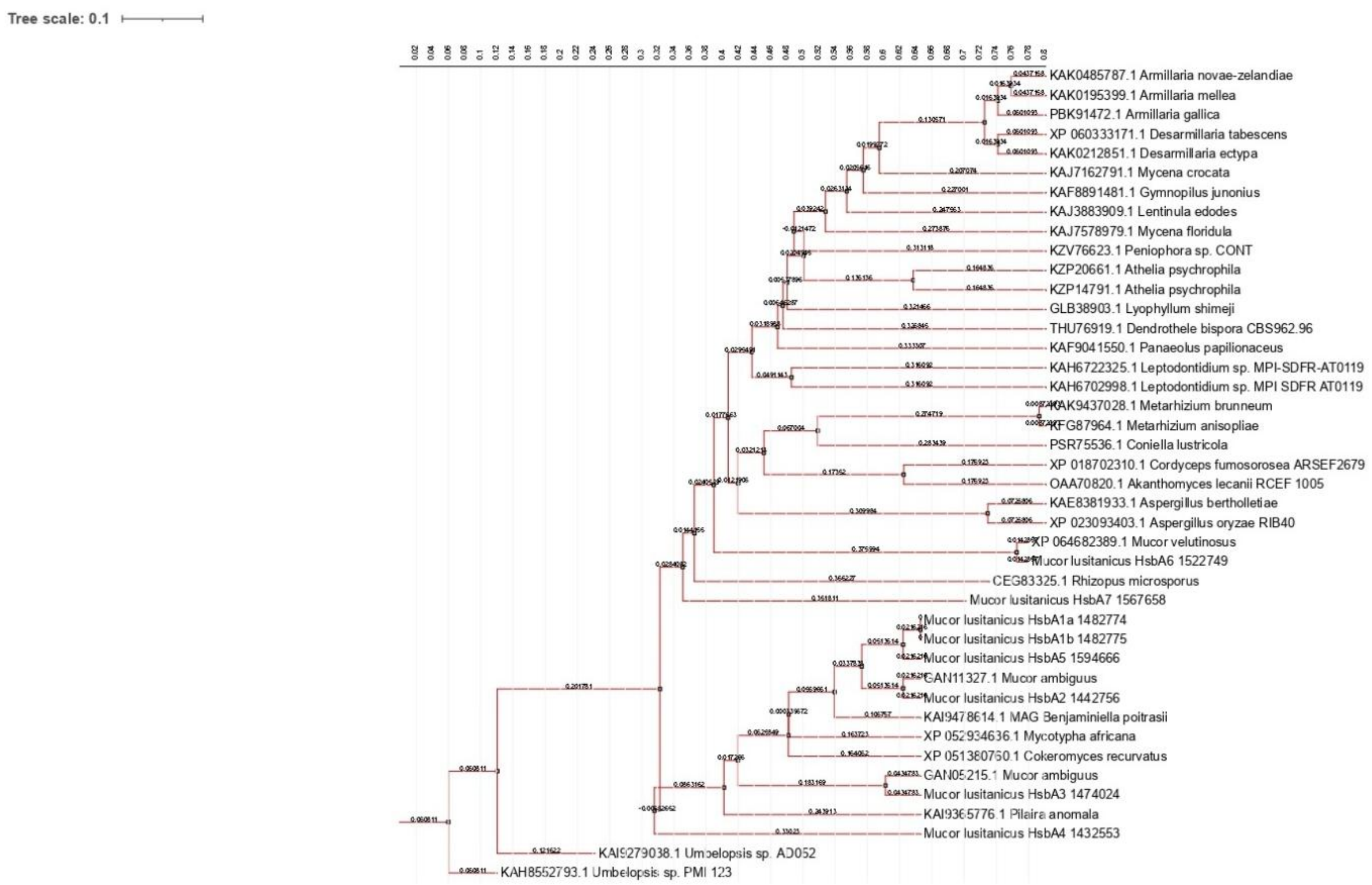

Supplementary Figure 2. Relative phylogenetic relationships of proteins showing >30% sequence similarity with *M. lusitanicus* HsbA proteins. The dendrogram was generated from Clustal Omega sequence alignment and visualized using iTOL v7 (Letunic and Bork, 2024). Branch lengths are indicated.

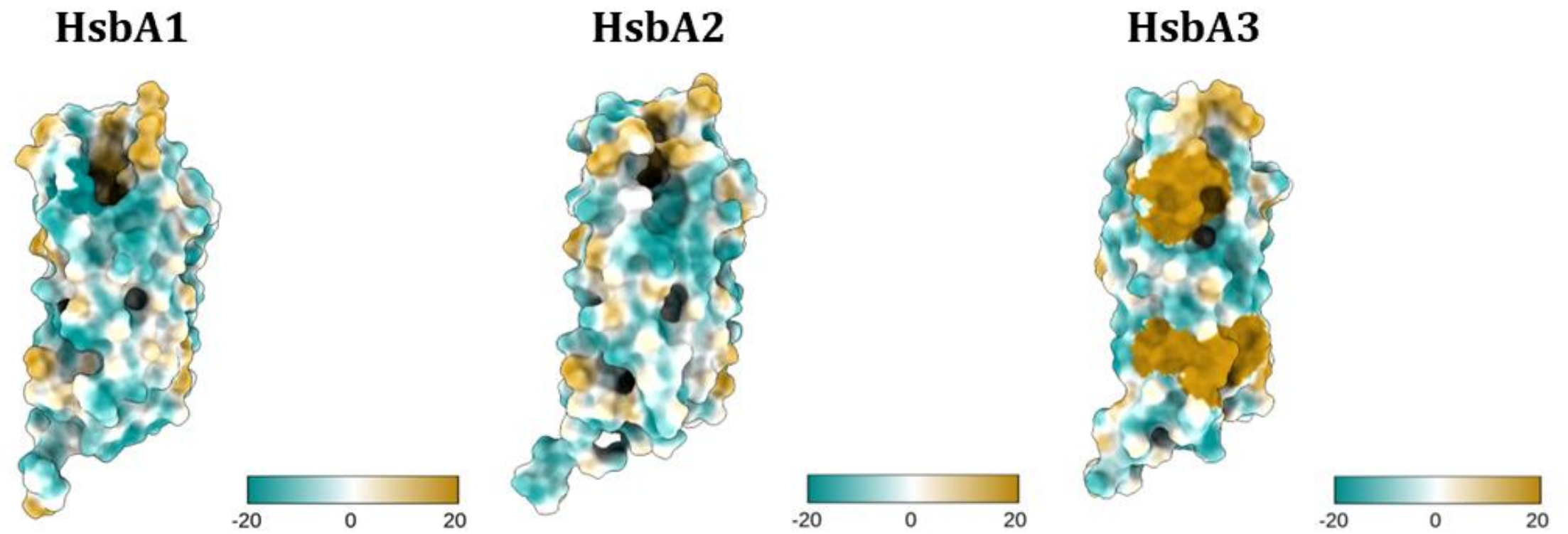

Supplementary Figure 3. Visualization of hydrophobic (yellow) and hydrophilic (cornflower blue) surface patches in the final molecular dynamics conformations of HsbA1–HsbA3 proteins generated using UCSF ChimeraX (Pettersen et al., 2021).

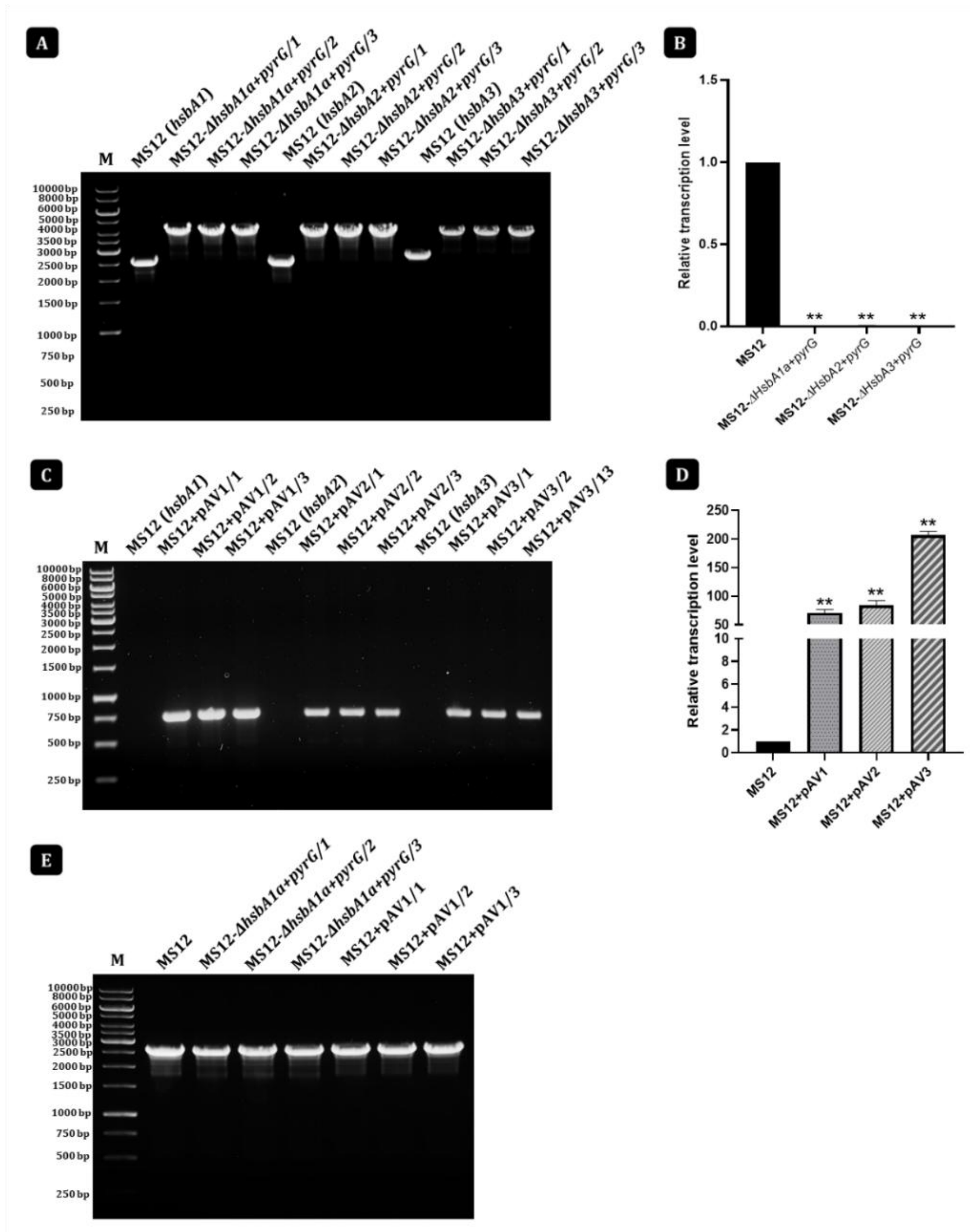

Supplementary Figure 4. (A) PCR analysis of the knock-out transformants. Agarose gel electrophoresis image of PCR products obtained with the promoter forward and terminator reverse primers; PCR was carried out with the primer pairs *McHsbA1a/1-McHsbA1a/6*, *McHsbA2/1-McHsbA2/6* and *McHsbA3/1-McHsbA3/4* for MS12-*ΔhsbA1a+pyrG*, MS12-*ΔhsbA2+pyrG* and MS12-*ΔhsbA3+pyrG*. **(B)** Relative transcription level of *hsbA1*, *hsbA2* and *hsbA3* in their respective deletion mutants and parental strain (MS12); **(C)** Confirmation of the presence of the plasmids pAV1, pAV2 and pAV3 in *M. lusitanicus* transformants. PCR was carried out with the primer pairs *Gpdfw-McHsbA1oerev*, *Gpdfw-McHsbA2oerev* and *Gpdfw-McHsbA3oerev* for MS12+pAV1, MS12+pAV2 and MS12+pAV3, respectively; **(D)** Relative transcription level of *hsbA1*, *hsbA2* and *hsbA3* in their respective overexpression mutants and parental strain (MS12). For relative expression level analyses strains were grown on YNB at 25 °C for 4 days; transcript level of each gene measured in the MS12 strain was taken as 1. The presented values are averages of three independent experiments; error bars indicate standard deviation. Values indicated by asterisks significantly differed from the value taken as 1 according to the statistical method unpaired t test (**: $p < 0.01$-0.0001). **(E)** Targeted disruption of

hsbA1a and copy-specific PCR confirmation of *hsbA1b* gene with promoter-terminator regions (2656 bp) variants in MS12 parental strain, MS12-*ΔhsbA1a+pyrG* and MS12+pAV1 mutants.

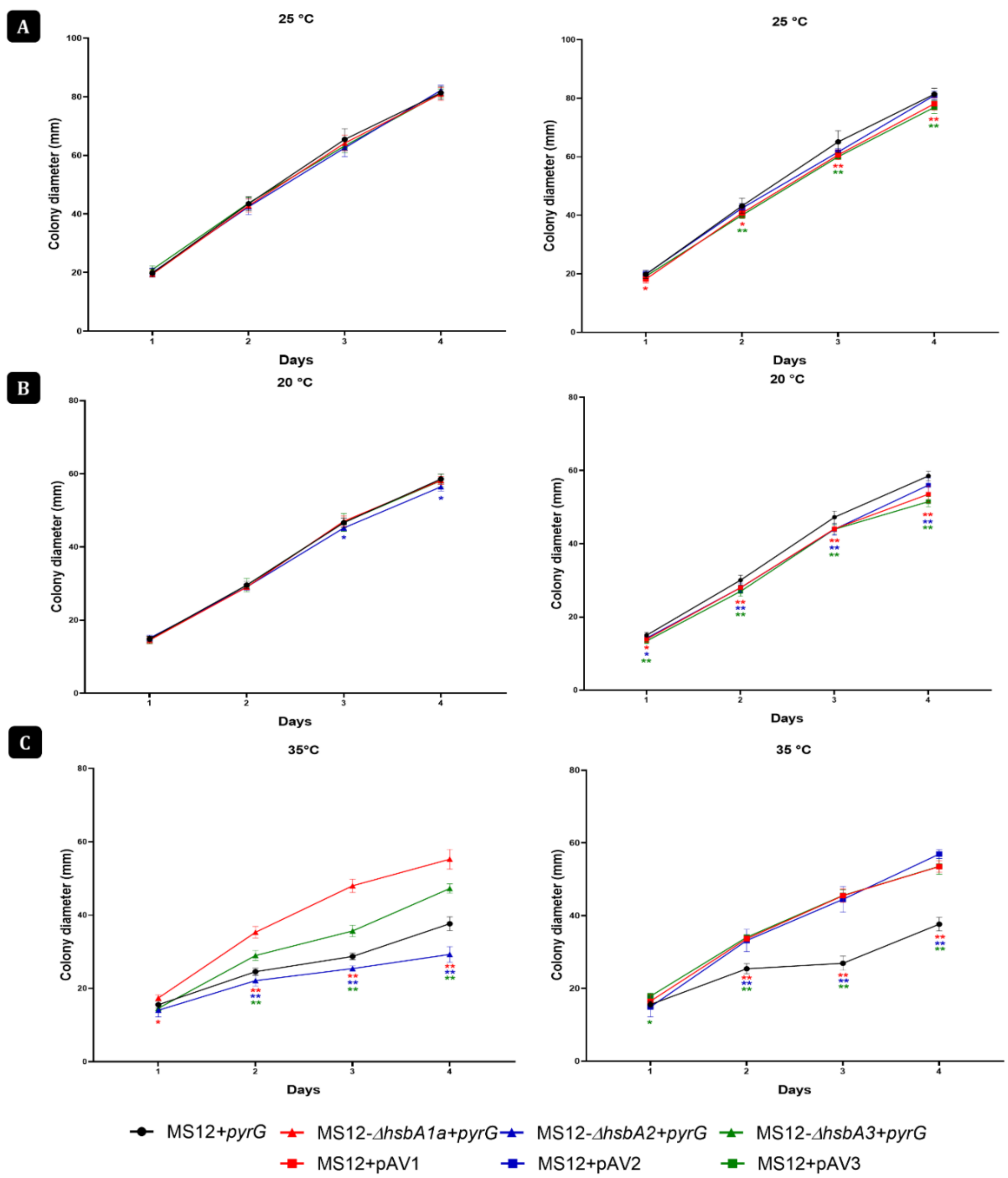

Supplementary Figure 5. Growth of *hsbA* mutants at different temperatures. Colony diameters were measured over a period of 4 days at the optimal growth temperature for the fungus (25 °C) **(A)** as well as at **(B)** 20 °C, **(C)** 35 °C); **(B)** effect of the lower temperature on growth of *hsbA* mutant strains on minimal medium for 4 days at 20 °C; **(C)** effect of the higher temperature on growth of *hsbA* mutant strains on minimal medium for 4 days at 35 °C. The values presented are from three independent cultivations (error bars indicate standard deviation). Values indicated by asterisks are significantly different from the value of the MS12+*pyrG* strain measured on the same day according to two-sample paired t test: *: p≤0.05-0.01; **: p≤0.001-0.0001. Error bars indicate standard deviation (SD).

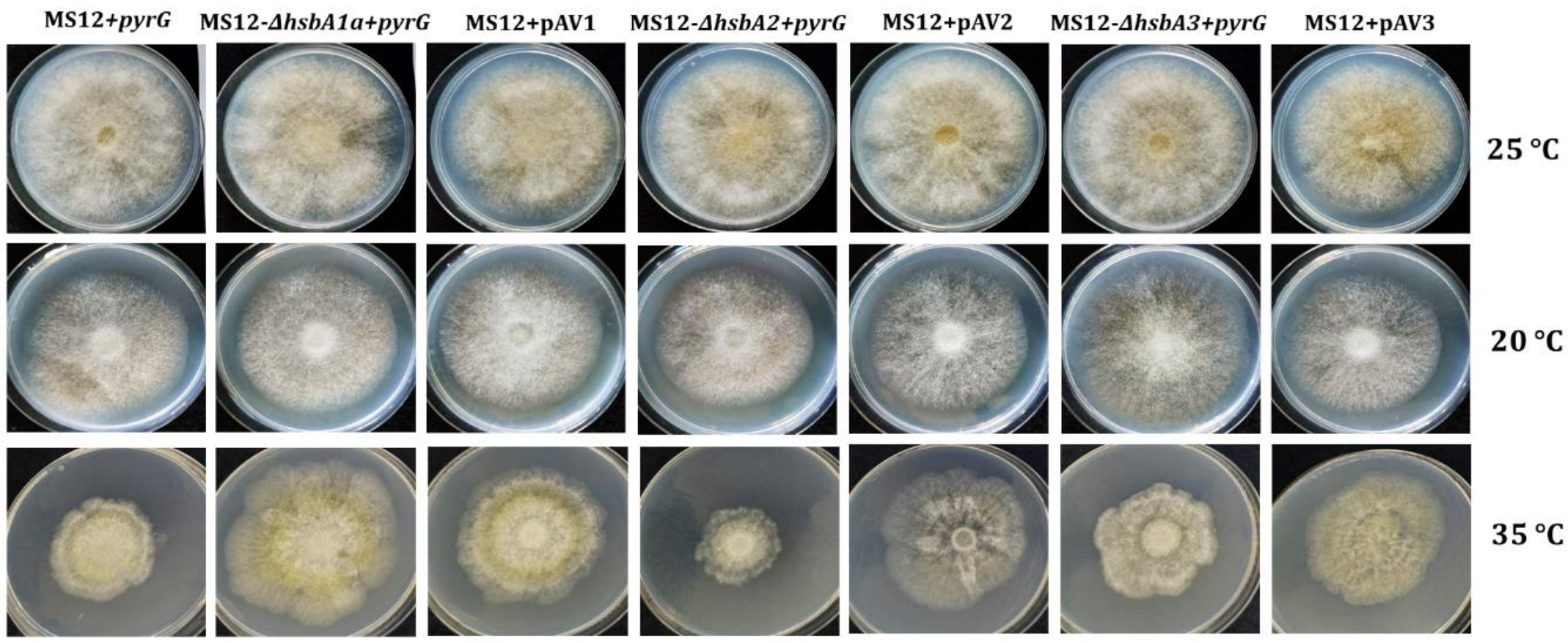

Supplementary Figure 6. Colony morphology of *hsbA* mutant strains. Colony morphology of *hsbA* mutants on YNB+L medium under aerobic conditions at different temperatures (25 °C; 20 °C; 35 °C) on the 4th day of cultivation.

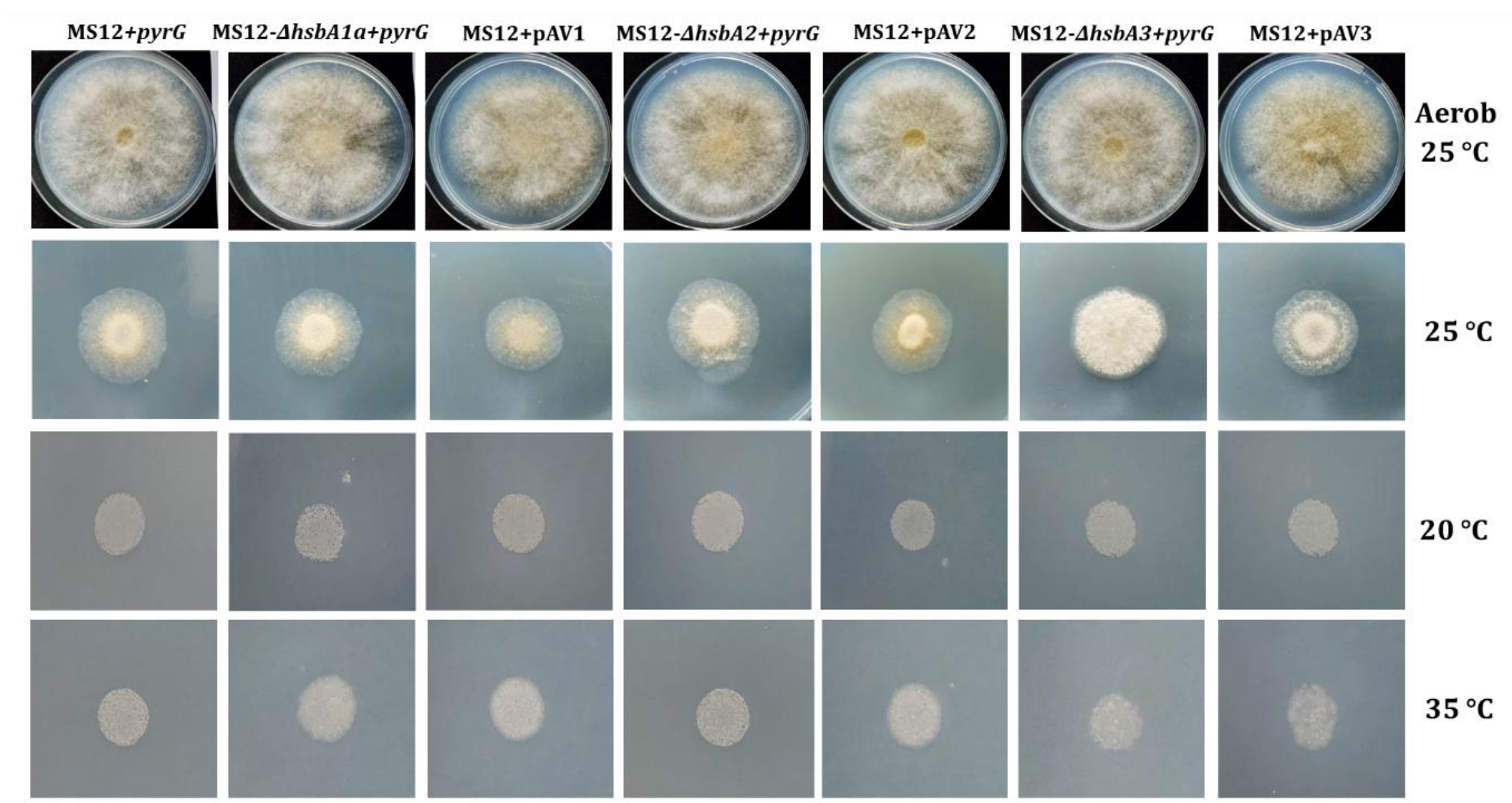

Supplementary Figure 7. Colony morphology of *hsbA* mutant strains. Colony morphology of *hsbA* mutants on YNB+L medium under anaerobic conditions at different temperatures (25 °C; 20 ° C; 35 °C) on the 4th day of cultivation.

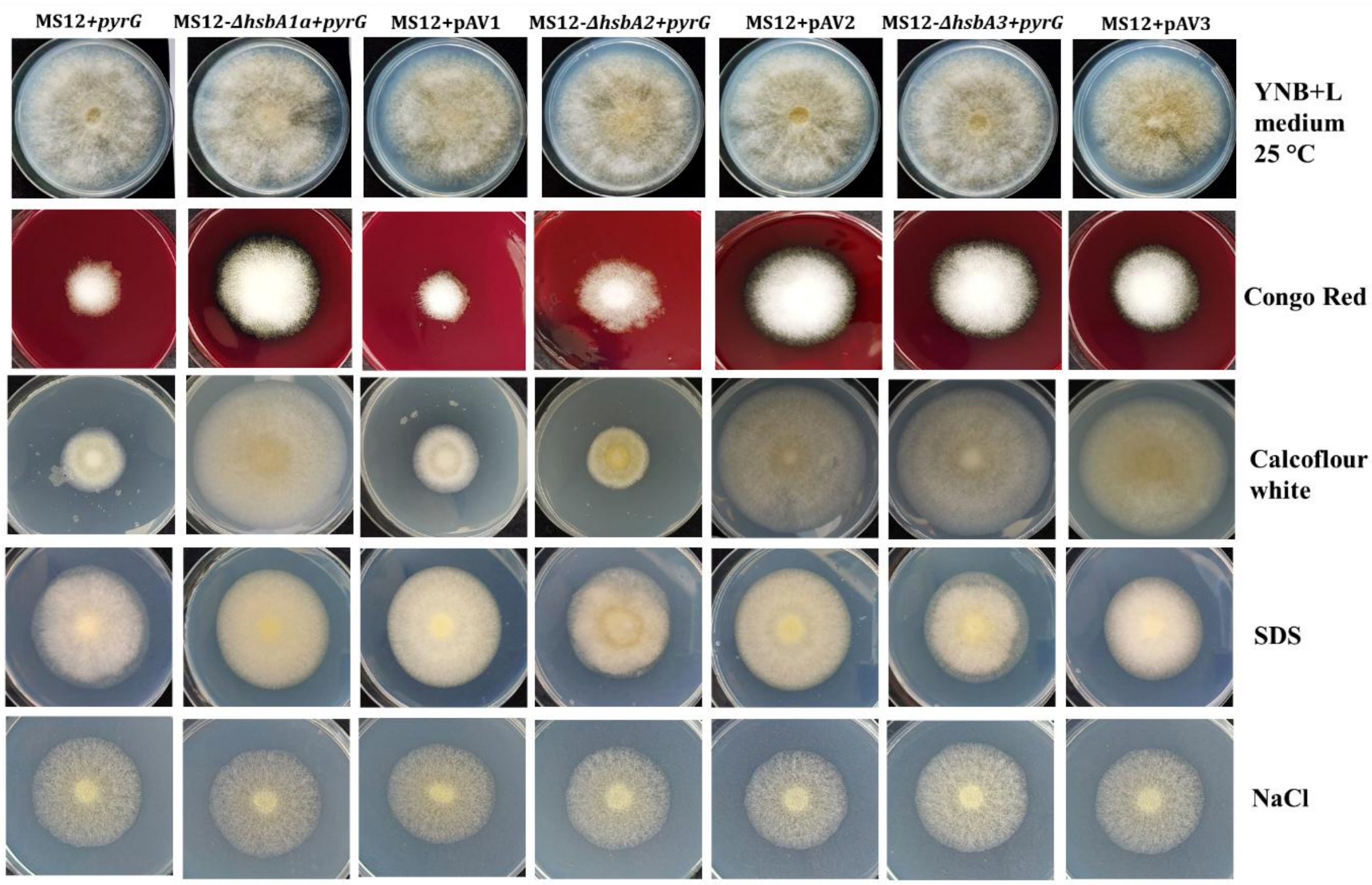

Supplementary Figure 8. Colony morphology of the *hsbA* mutants under different stress conditions. Effect of Congo Red (CR), Calcofluor White (CFW; a cell wall stressor), SDS (a membrane stressor), and NaCl (an osmotic stressor) on the growth of *hsbA* mutant strains.

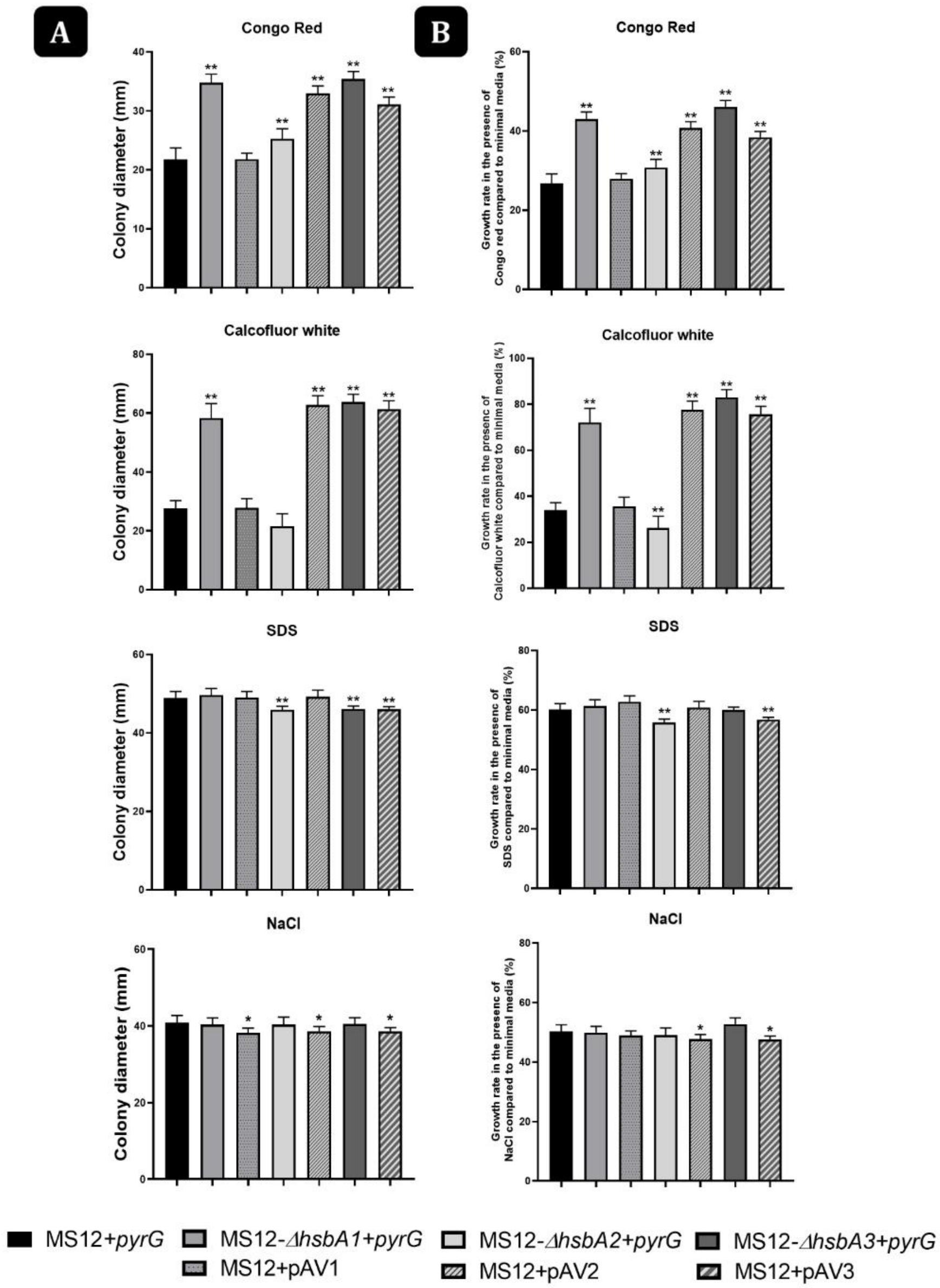

Supplementary Figure 9. Effect of different stressors on *hsbA* mutants. **(A)** Effect of Congo Red (CR), Calcofluor White (CFW; a cell wall stressor), SDS (a membrane stressor), and NaCl (an osmotic stressor) on the growth of *hsbA* mutant strains. The impact of each stressor was assessed by comparing colony diameters measured under control conditions and, in the presence of the stressor on day 4 of cultivation. The values represent measurements from three independent cultivations. **(B)** Growth rate in percentages (%) in the presence of stressors compared to minimal media at 25 °C and on the 4th day of cultivation. Error bars indicate standard deviation (SD). Values marked with asterisks are significantly different from the corresponding value of the MS12+*pyrG* strain based on a two-sample unpaired t test: *: p≤0.05-0.01; **: p≤0.001-0.0001.

1.2 Supplementary Tables

Supplementary Table 1: Details of primers used for qRT-PCR, gene disruption, and overexpression of hsbA mutant strains.

Name of the primer	Sequence 5'-3'	Name of the amplicon/used for	Length of the amplicon (bp)
**Primers used for qRT-PCR**			
MchsbA1_RT1	CCCGAGTTTGATGCCATTCT	segment of *hsbA1* gene coding sequence	159
MchsbA1_RT2	GGAGTTGTTGATTTGAGTGACCA		
MchsbA2_RT1	TCAACACTGTAAGCACCCTTG	segment of *hsbA2* gene coding sequence	192
MchsbA2_RT2	CAGCATCCAAATGAGTAGCGG		
MchsbA3_RT1	CTTACTATTGCTGCCTCAGTCA	segment of *hsbA3* gene coding sequence	197
MchsbA3_RT2	GTTTCAAGAGTTTGTTCACTGGAG		
McActinF	CACTCCTTCACTACCACCGCT	segment of *actin* (ACT) gene coding sequence	117
McActinR	GAGAGCAGAGGATTGAGCAGC		
**Primers designed to disrupt the *hsbA1a* gene**			
MchsbA1a/1	AATGTGCTGTTGGTTACACGA	*hsbA1* promoter region	1109
MchsbA1a/2	CAGAGTTGATGTCGTCAATACAG		
MchsbA1a/3	GTCCAACTCTGTATTGACGACATCAACTCTGTGCCTCAGCATTGGTACTTG	*pyrG* gene with its own promoter and terminator region	2055
MchsbA1a/4	CTTGGTTTGGGTATCCAAGTTCTTGATGTCGTACACTGGCCATGCTATCG		
MchsbA1a/5	GACATCAAGAACTTGGATACCC	*hsbA1* terminator region	1129
MchsbA1a/6	GGTTTGGACATCTCAACACCT		
MchsbA1a/7	GTACTCTTCCATACATTCCATTCTG	nested primers	3864
MchsbA1a/8	AAGCGCATAGTAAACTGACC		
**Primers designed to disrupt the *hsbA2* gene**			
MchsbA2/1	AAATAGCCTGAGCAGTTGTTTGAG	*hsbA2* promoter region	1108
MchsbA2/2	GGCTTTAACAATGGCAAGTTGAG		
MchsbA2/3	GCTGCTGCTCAACTTGCCATTGTTAAAGCCTGCCTCAGCATTGGTACTTG	*pyrG* gene with its own promoter and terminator region	2065
MchsbA2/4	TGGTTTGAGAGTCCAGGTTCTTGATGTCACGTACACTGGCCATGCTATCG		
MchsbA2/5	GTGACATCAAGAACCTGGAC	*hsbA2* terminator region	1138
MchsbA2/6	TGAATGACAAGATACACGCC		
MchsbA2/7	AATGGCCAATGGTATTAGTTCCC	nested primers	3891

MchsbA2/8	AGCTATAATCTGTACCGCACTG		
**Primers designed to disrupt the *hsbA3* gene**			
MchsbA3/1	CAAACGAATGTTGGGATTTCTC	*hsbA3* promoter region	1155
MchsbA3/2	TTTGATTTGATTTGTTTGTTGTTG A		
MchsbA3/3	GTTAGCATAGCTAGTCTTTATCC CT	*hsbA3* terminator region	1106
MchsbA3/4	CATGGTACAAATGGCTTACGG		
MchsbA3/5	CAATCAAATCTCAACAACAAACA AATCAAATCAAATGCCTCAGCAT TGGTACTTG	*pyrG* gene with its own promoter and terminator region	2065
MchsbA3/6	CATGGCAAGTAGGGATAAAGAC TAGCTATGCTAACGTACACTGGC CATGCTATCG		
MchsbA3/7	CTCCGCCCTATTAAAGAAGCA	nested primers	3829
MchsbA3/8	ACTTATGCCAAATGTCGTGAG		
**Primers designed to overexpress HsbA proteins**			
MchsbA1oe fw	TTCCTCGAGATGCGTGCCTTCTCC ACTTTAATC	*hsbA1* gene with restriction site for the construction of pAV1 overexpression plasmid	640
MchsbAoe rev	TTCATCGATTTAGATACCATAAG CGGTCTTGGC		
MchsbA2oe fw	TTCCTCGAGATGCGCGCTTTC TCTACATTACTC	*hsbA2* gene with restriction site for the construction of pAV2 overexpression plasmid	636
MchsbA2oe rev	TTCATCGAT TTAGATACCATAAGCGGTCTTGG C		
MchsbA3oe fw	TTCCTCGAGATGCGCGCCTCTCT CTCTTT	*hsbA3* gene with restriction site for the construction of pAV3 overexpression plasmid	573
MchsbA3oe rev	TTCATCGATTTAAATACCGTAAG CGGTCTTGGCAGAGGC		
Gpd fw	CATGAAGTGTGAGACATTGCGA	*gpd* promoter for the analysis of the overexpressed mutants	-
**Primers designed to amplify the *hsbA1b* gene**			
MchsbA1b promoter fw	AGGGGAACATATCCAATTGGCCT C	*hsbA1b* gene with its own promoter and terminator region	2656
MShsbA1b terminator rev	CTGGGGCACAAACTGTGTCAGAT		

Supplementary Table 2: Proteins homologous to the different HsbA proteins of *M. lusitanicus* found by BLASTp search in the NCBI database.

Protein homologues (>30% identity)				
**Protein**	**Accession**	**Description**	**Organism**	**Identities (%)**
**HsbA1a/ HsbA1b**	GAN11327.1	hydrophobic surface binding protein	*Mucor ambiguus*	87.03%
	KAI9478614.1	MAG: hydrophobic surface binding protein A-domain-containing protein	*Benjaminiella poitrasii*	82.16%
	XP_051380760.1	hydrophobic surface binding protein A-domain-containing protein, partial	*Cokeromyces recurvatus*	73.62%
	XP_052934636.1	hydrophobic surface binding protein A-domain-containing protein	*Mycotypha africana*	71.17%
	GAN05215.1	hydrophobic surface binding protein	*Mucor ambiguus*	63.58%
	KAI9279038.1	hydrophobic surface binding protein A-domain-containing protein	*Umbelopsis sp. AD052*	46.88%
	KAH8552793.1	hydrophobic surface binding protein A-domain-containing protein	*Umbelopsis sp. PMI_123*	42.50%
	KAK0485787.1	hydrophobic surface binding protein	*Armillaria novae-zelandiae*	34.67%
	XP_060333171.1	hydrophobic surface binding protein	*Desarmillaria tabescens*	34.67%
	KZP20661.1	hydrophobic surface binding protein	*Athelia psychrophila*	34.46%
	PBK91472.1	hydrophobic surface binding protein	*Armillaria gallica*	34.00%
	KAJ7578979.1	hydrophobic surface binding protein	*Mycena floridula*	33.56%
	KAK0212851.1	hydrophobic surface binding protein	*Desarmillaria ectypa*	32.67%
	XP_018702310.1	Cell wall galactomannoprotein	*Cordyceps fumosorosea ARSEF 2679*	32.10%
	ATY65847.1	Cell wall galactomanno	*Cordyceps militaris*	31.48%
	KAK0195399.1	hydrophobic surface binding protein	*Armillaria mellea*	31.45%
	GLB38903.1	putative hydrophobic surface binding protein	*Lyophyllum shimeji*	31.33%
	OAA70820.1	Cell wall galactomannoprotein	*Akanthomyces lecanii RCEF 1005*	31.03%
	KAH6722325.1	hydrophobic surface binding protein A-domain-containing protein	*Leptodontidium sp. MPI-SDFR-AT-0119*	30.92%
	KAE8381933.1	hydrophobic surface binding protein A-domain-containing protein	*Aspergillus bertholletiae*	30.00%
**HsbA2**	GAN11327.1	hydrophobic surface binding protein	*Mucor ambiguus*	95.71%
	KAI9478614.1	MAG: hydrophobic surface binding protein A-domain-containing protein	*Benjaminiella poitrasii*	75.93%
	XP_052934636.1	hydrophobic surface binding protein A-domain-containing protein	*Mycotypha africana*	73.01%
	XP_051380760.1	hydrophobic surface binding protein A-domain-containing protein, partial	*Cokeromyces recurvatus*	71.17%
	GAN05215.1	hydrophobic surface binding protein	*Mucor ambiguus*	58.64%
	KAI9279038.1	hydrophobic surface binding protein A-domain-containing protein	*Umbelopsis sp. AD052*	47.50%

	KAH8552793.1	hydrophobic surface binding protein A-domain-containing protein	*Umbelopsis sp. PMI_123*	45.00%
	XP_060333171.1	hydrophobic surface binding protein	*Desarmillaria tabescens*	36.00%
	KAJ7769452.1	hydrophobic surface binding protein	*Mycena metata*	34.00%
	PSR75536.1	antigenic cell wall galactomanno protein-like protein, partial	*Coniella lustricola*	33.33%
	KZV76623.1	hydrophobic surface binding protein	*Peniophora sp. CONT*	32.89%
	KAJ3883909.1	hydrophobic surface binding protein	*Lentinula edodes*	32.67%
	GLB38903.1	putative hydrophobic surface binding protein	*Lyophyllum shimeji*	32.45%
	KAF9041550.1	hydrophobic surface binding protein A-domain-containing protein	*Panaeolus papilionaceus*	31.61%
	KAJ7578979.1	hydrophobic surface binding protein	*Mycena floridula*	31.10%
	KAK9437028.1	Cell wall galactomannoprotein	*Metarhizium brunneum*	31.08%
	KFG87964.1	putative antigenic cell wall galactomannoprotein	*Metarhizium anisopliae*	31.08%
	XP_023093403.1	cell wall mannoprotein 1 family protein	*Aspergillus oryzae RIB40*	31.08%
	XP_001882714.1	hydrophobic surface binding protein	*Laccaria bicolor S238N-H82*	30.95%
	KAJ1714944.1	antigenic cell wall galactomannoprotein	*Aspergillus flavus*	30.86%
	KAB8252746.1	hydrophobic surface binding protein A-domain-containing protein	*Aspergillus flavus*	30.86%
	KAB8209297.1	hydrophobic surface binding protein A-domain-containing protein	*Aspergillus parasiticus*	30.86%
	XP_015409780.1	cell wall mannoprotein 1 family protein	*Aspergillus nomiae NRRL 13137*	30.86%
	XP_007824124.1	antigenic cell wall galactomannoprotein	*Metarhizium robertsii ARSEF 23*	30.41%
	XP_031936334.1	cell wall mannoprotein 1 family protein	*Aspergillus pseudonomiae*	30.29%
	KAF9524354.1	hydrophobic surface binding protein A-domain-containing protein	*Crepidotus variabilis*	30.20%
	XP_022388462.1	cell wall mannoprotein 1 family protein	*Aspergillus bombycis*	30.06%
	KOC12680.1	antigenic cell wall galactomannoprotein	*Aspergillus flavus AF70*	30.06%
	XP_031919806.1	cell wall mannoprotein 1 family protein	*Aspergillus pseudotamarii*	30.06%
	XP_041149948.1	putative antigenic cell wall galactomanno protein	*Aspergillus flavus*	30.06%
**HsbA3**	GAN05215.1	hydrophobic surface binding protein	*Mucor ambiguus*	91.30%

	GAN11327.1	hydrophobic surface binding protein	*Mucor ambiguus*	58.01%
	KAI9478614.1	MAG: hydrophobic surface binding protein A-domain-containing protein	*Benjaminiella poitrasii*	57.84%
	XP_052934636.1	hydrophobic surface binding protein A-domain-containing protein	*Mycotypha africana*	55.98%
	XP_051380760.1	hydrophobic surface binding protein A-domain-containing protein, partial	*Cokeromyces recurvatus*	54.34%
	KAI9279038.1	hydrophobic surface binding protein A-domain-containing protein	*Umbelopsis sp. AD052*	47.22%
	KAH8552793.1	hydrophobic surface binding protein A-domain-containing protein	*Umbelopsis sp. PMI_123*	43.18%
	KZP20661.1	hydrophobic surface binding protein	*Athelia psychrophila*	38.33%
	CEG83325.1	Putative STE/STE20/PAKA protein kinase	*Rhizopus microsporus*	37.36%
	KAJ3883909.1	hydrophobic surface binding protein	*Lentinula edodes*	34.07%
	KAK0212851.1	hydrophobic surface binding protein	*Desarmillaria ectypa*	33.71%
	KAH6702998.1	hydrophobic surface binding protein A-domain-containing protein	*Leptodontidium sp. MPI-SDFR-AT-0119*	33.33%
	KAK0195399.1	hydrophobic surface binding protein	*Armillaria mellea*	33.16%
	KAK0485787.1	hydrophobic surface binding protein	*Armillaria novae-zelandiae*	33.15%
	PBK91472.1	hydrophobic surface binding protein	*Armillaria gallica*	32.58%
	GLB38903.1	putative hydrophobic surface binding protein	*Lyophyllum shimeji*	31.47%
	KAF8891481.1	hydrophobic surface binding protein A-domain-containing protein	*Gymnopilus junonius*	31.11%
	TDL2 3474.1	hydrophobic surface binding protein	*Rickenella mellea*	30.94%
	KAF5324335.1	hypothetical protein D9619_011311	*Psilocybe cf. subviscida*	30.86%
	KAH7102528.1	hydrophobic surface binding protein	*Auriculariales sp. MPI-PUGE-AT-0066*	30.56%
	KAF7343844.1	Hydrophobic surface binding protein	*Mycena sanguinolenta*	30.56%
	KAJ7162791.1	hydrophobic surface binding protein	*Mycena crocata*	30.34%
**HsbA4**	GAN05215.1	hydrophobic surface binding protein	*Mucor ambiguus*	42.39%
	KAH8552793.1	hydrophobic surface binding protein A-domain-containing protein	*Umbelopsis sp. PMI_123*	31.15%
	KAI9279038.1	hydrophobic surface binding protein A-domain-containing protein	*Umbelopsis sp. AD052*	31.11%
	KAH9217354.1	hydrophobic surface binding protein A-domain-containing protein	*Leptodontidium sp. 2 PMI_412*	30.00%
**HsbA5**	GAN11327.1	hydrophobic surface binding protein	*Mucor ambiguus*	86.50%
	KAI9478614.1	MAG: hydrophobic surface binding protein A-domain-containing protein	*Benjaminiella poitrasii*	80.25%
	XP_051380760.1	hydrophobic surface binding protein A-domain-containing protein, partial	*Cokeromyces recurvatus*	73.01%

	XP_052934636.1	hydrophobic surface binding protein A-domain-containing protein	*Mycotypha africana*	71.78%
	KAI9279038.1	hydrophobic surface binding protein A-domain-containing protein	*Umbelopsis sp. AD052*	46.25%
	KAH8552793.1	hydrophobic surface binding protein A-domain-containing protein	*Umbelopsis sp. PMI_123*	41.25%
	PSR75536.1	antigenic cell wall galactomanno protein-like protein, partial	*Coniella lustricola*	34.34%
	THU76919.1	hydrophobic surface binding protein	*Dendrothele bispora CBS 962.96*	33.73%
	XP_018702310.1	Cell wall galactomannoprotein	*Cordyceps fumosorosea ARSEF 2679*	32.10%
	GLB38903.1	putative hydrophobic surface binding protein	*Lyophyllum shimeji*	32.00%
	KZV76623.1	hydrophobic surface binding protein	*Peniophora sp. CONT*	31.54%
	OAA70820.1	Cell wall galactomannoprotein	*Akanthomyces lecanii RCEF 1005*	31.03%
	ATY65847.1	Cell wall galactomanno	*Cordyceps militaris*	30.25%
	KAL6817229.1	hydrophobic surface binding protein A domain-containing protein	*Trichoderma sp. SZMC 28015*	30.10%
	KAL7911835.1	hydrophobic surface binding protein A domain-containing protein	*Trichoderma velutinum*	30.10%
**HsbA6**	XP_064682389.1	tRNA (guanine(9)-N(1))-methyltransferase	*Mucor velutinosus*	97.14%
**HsbA7**	GAN11327.1	hydrophobic surface binding protein	*Mucor ambiguus*	47.27%
	KAI9478614.1	MAG: hydrophobic surface binding protein A-domain-containing protein	*Benjaminiella poitrasii*	46.99%
	XP_052934636.1	hydrophobic surface binding protein A-domain-containing protein	*Mycotypha africana*	46.34%

Supplementary Table 3: The number of all identified variants in the strains.

Sample name	Number of SNPs	Number of indels
MS12	293	51
MS12-*ΔhsbA1a+pyrG*	290	46
MS12-*ΔhsbA2+pyrG*	302	43

Supplementary Table 4: Expression patterns of *hsbA1-3* genes in various mutant strains.

Name of the strain	*hsbA1 expression*	*hsbA2 expression*	*hsbA3 expression*
**MS12-*ΔhsbA1a+pyrG***	absent	upregulated	no change
**MS12-*ΔhsbA2+pyrG***	no change	absent	no change
**MS12-*ΔhsbA3+pyrG***	no change	upregulated	absent
**MS12+pAV1**	overexpressed	upregulated	no change
**MS12+pAV2**	upregulated	overexpressed	upregulated
**MS12+pAV3**	no change	upregulated	overexpressed
